# Health and Wellness Technology Use by Historically Underserved Health Consumers: Systematic Review

**DOI:** 10.2196/jmir.2095

**Published:** 2012-05-31

**Authors:** Enid Montague, Jennifer Perchonok

**Affiliations:** ^1^Department of Industrial and Systems EngineeringUniversity of Wisconsin-MadisonMadison, WIUnited States

**Keywords:** health care disparities, biomedical technology, health education, health knowledge, attitudes, and practice, health care quality, access, and evaluation, educational technology, cultural diversity

## Abstract

**Background:**

The implementation of health technology is a national priority in the United States and widely discussed in the literature. However, literature about the use of this technology by historically underserved populations is limited. Information on culturally informed health and wellness technology and the use of these technologies to reduce health disparities facing historically underserved populations in the United States is sparse in the literature.

**Objective:**

To examine ways in which technology is being used by historically underserved populations to decrease health disparities through facilitating or improving health care access and health and wellness outcomes.

**Methods:**

We conducted a systematic review in four library databases (PubMed, PsycINFO, Web of Science, and Engineering Village) to investigate the use of technology by historically underserved populations. Search strings consisted of three topics (eg, technology, historically underserved populations, and health).

**Results:**

A total of 424 search phrases applied in the four databases returned 16,108 papers. After review, 125 papers met the selection criteria. Within the selected papers, 30 types of technology, 19 historically underserved groups, and 23 health issues were discussed. Further, almost half of the papers (62 papers) examined the use of technology to create effective and culturally informed interventions or educational tools. Finally, 12 evaluation techniques were used to assess the technology.

**Conclusions:**

While the reviewed studies show how technology can be used to positively affect the health of historically underserved populations, the technology must be tailored toward the intended population, as personally relevant and contextually situated health technology is more likely than broader technology to create behavior changes. Social media, cell phones, and videotapes are types of technology that should be used more often in the future. Further, culturally informed health information technology should be used more for chronic diseases and disease management, as it is an innovative way to provide holistic care and reminders to otherwise underserved populations. Additionally, design processes should be stated regularly so that best practices can be created. Finally, the evaluation process should be standardized to create a benchmark for culturally informed health information technology.

## Introduction

While the visibility of health disparities has recently come to the forefront of the US health care agenda, the topic of health care disparities is not new. In 1984 the health of the nation was addressed in the “Health, United States, 1983” report conducted by the US Department of Health and Human Services. This report stated that African Americans and other racial and ethnic minorities were experiencing a higher burden of death and illness than the rest of the nation [1]. As a reaction to this report, the Secretary of the Department of Health and Human Services created the first group solely designated to study minority health issues—the Task Force on Black and Minority Health. In 1985, this group published a comprehensive study on minority health problems, “Report of the Secretary’s Task Force on Black and Minority Health.” The report brought more awareness of health disparities in historically underserved populations and spurred research [1].

In the United States, historically underserved populations are growing in size, and hence health disparities are affecting a growing proportion of Americans. For instance, while 2000 census findings showed that 82% of the population was white, by 2015 this number is predicted to decrease to 79%. At that time, it is expected that these will be 5% Asian, 13% African American, and 15% Latino [2]. By 2050, ethnic populations will double in size in the United States and constitute 40% of the population [2]. Similar studies conducted outside of the census found similar results. Partida reported that one in eight Americans is foreign born, and 45% of children less than 5 years of age are not white [3]. Beyond ethnicity, the percentage of older Americans is also increasing, with the oldest (85+ years of age) and ethnic elderly populations growing at the fastest rates [4].

With historically underserved populations growing in the United States, it is important to study the potential and existing health disparities facing them. While there is no consensus regarding the specific definition of what constitutes a health disparity, the National Institutes of Health defined a health disparity as “differences in the incidence, prevalence, mortality, and burden of diseases and other adverse health conditions that exist among specific population groups in the United States” [5]. More specifically health disparities include inequalities and inequities in (1) environment, (2) access to, use of, and quality of care, (3) health status, or (4) a specific health outcome [5]. Examples of racial and ethnic health disparities include certain populations with exceedingly high rates of cardiovascular disease, diabetes, asthma, and cancer [6]. While factors including lower socioeconomic status, lack of insurance, lower levels of education, and living in communities with more environmental hazards have been cited as social determinants outside of the health care system, these do not fully account for health disparities [6]. A patient’s culture may contribute to the disparities facing them by influencing health beliefs, values, preferences, and behaviors. For instance, a patient’s ability to recognize symptoms and then effectively describe the symptoms to a provider will influence their interactions with their provider, which can in turn affect their health outcomes [6]. The United States made eliminating health disparities one of its main goals through Healthy People 2010, a federal interagency workgroup that provides 10-year national objectives for improving the general health of Americans [7]. Healthy People 2010 targeted disparities based on race and ethnicity, gender, education, income, geographic location, disability status, or sexual orientation [8].

As technology is being used to further the success of the health care system, there is interest in understanding how the increased use of technology affects the already unequal ability of minorities to access health care [9]. Health technology has been used since as early as the middle to late 19th century, when electrocardiograph data were transmitted over telephone wires [10]. Today, health information technology (IT) is used to benefit both the health care consumer and public health as a whole. Health care consumers benefit from health IT by receiving a higher quality of care, reduction in medical errors, fewer duplicate treatments and tests, decreases in paperwork, lower health care costs, access to health information, and access to affordable care [11]. The public health sector benefits from health IT, as it can facilitate earlier detection of infectious disease outbreaks, improve the tracking of chronic disease management, and gather de-identified data for research [11].

Technology can be used in a variety of ways to positively affect historically underserved health care consumers. For example, telemedicine has been suggested as a possible way to address health care disparities among historically underserved urban populations. Research shows that urban communities are often unable to access health care in a timely manner due to low physician-to-population ratios, limited specialty care, and overcrowded, inadequate, and inefficient organizational structures [12]. Telemedicine is an innovative way to decrease the health care gap through mitigating geographic barriers [12].

To promote widespread adoption of health IT, the US Department of Health and Human Services established the Office of the National Coordinator for Health Information Technology [13]. Health IT refers to “a variety of electronic methods used to manage information about people’s health and health care, on both an individual and a group level” [14]. Research has shown that health IT can enhance quality, communication [15], and cost-effective care [16], and can facilitate culturally competent outreach and education [17].

The purpose of this review was to examine ways in which technology is being used by historically underserved populations in order to decrease the health disparity through facilitating or improving health care access and health and wellness outcomes. While several studies have investigated how historically underserved populations use technology when addressing their health, these studies focused on a single historically underserved group or a single health issue. We used a methods-description approach method to synthesize published research from reference databases to draw a larger conclusion from the current literature [18]. We explored four main questions from the reviewed papers. (1) Which types of technologies are used to address potential disparities? (2) Which health issues are addressed in the reviewed papers? (3) Which historically underserved groups are targeted for technology-based interventions? (4) How are the health benefits and technologies evaluated? The systematic review was conducted in four reference databases (PubMed, PsycINFO, Web of Science, and Engineering Village) with search strings consisting of three topics: technology, historically underserved groups, and health. Findings are divided into five sections, each answering one of the five main questions. Outcomes include recommendations for increased use of certain technology, along with recommendations to use culturally informed technology in regard to distinct types of health conditions.

## Methods

### Definitions

The term minority has been used often in health research. However, the term is problematic, as it can create a sense of inferiority for the population in question [19]. Eysenbach suggested that eHealth is a broad topic encompassing 10 main concepts, one of which is equity [20]. He noted that certain patient populations are disadvantaged based on their lack of money, skills, and access to computers. However, the use of the term minority only further perpetrates these inequities. For this reason, we will not use the term minority in this paper. Instead, we will use the term historically underserved to refer to populations that are disadvantaged based on their race, ethnicity, age, gender, socioeconomic status, health status, or geographic location.

Larson stated that simplistic definitions of health should be avoided, as they lead to simplistic measures of health, health outcomes, and quality of care [21]. Therefore, it is important to use a more holistic definition of health that includes wellness; for instance, the World Health Organization defined health as “a state of complete physical, mental and social well-being, not just absence of disease” [21]. Pervasive health care takes this concept a step further and can be defined as “healthcare to anyone, anytime, and anywhere by removing locational, time and other restraints while increasing both the coverage and the quality of healthcare” [22].

The *Health Technology Assessment Handbook *defined health technology as “a collective term for procedures and methods for examination, treatment, care and rehabilitation of patients, including instruments, drugs, and preventive procedures” [23]. Health IT, which differs slightly from health technology, refers to the implementation of information processing that deals with the storage, retrieval, sharing, and use of health care information, data, and knowledge to facilitate both decision making and communication [24]. Health IT used directly by consumers is called consumer health IT. Or and Karsh defined consumer health IT as “computer-based systems that are designed to facilitate information access and exchange, enhance decision making, provide social and emotional support, and help behavior changes that promote health and well-being” [25].

eHealth refers to the use of electronic communication and information technology within the health sector. Tools often referred to in connection with eHealth include personal digital assistants, compact discs and DVDs, and interactive games [26]. Telemedicine, which is a part of eHealth, allows providers and patients in different geographic locations to communicate through computers, information, and telecommunication [12]. Telehealth, which is often used synonymously with telemedicine, is defined by the World Health Organization as telemedicine used by others beyond the physician [10] such as nurses and pharmacists. For this review, the author of the original paper differentiated between these 2 terms. For instance, if the author of the paper under review used the term telemedicine, we used it for this review; if the author of the reviewed paper used telehealth, we used it for this review as well.

Electronic health records (EHRs) are an electronic form of the traditional patient health record (patient’s health profile, and environmental and behavioral information). EHRs include a time dimension and allow multiple providers to contribute information to the record [27]. EHRs have been shown to have a positive influence on quality of care, patient safety, and system delivery [17]. Electronic medical records are similar to EHRs except they are created solely for care delivery organizations—that is, hospitals and physician’s offices [28]. EHRs have the ability to increase access to health care, reduce medication errors, and improve administrative efficiency and quality of care [16]. In contrast to the EHR, personal health records are an optional tool that allows people to manage their own health records [29]. The personal health record is a lifelong resource of health information that is managed by the individual in an electronic, universally available form [29].

For this review, we used a broad definition of technology that includes technology designed for both health and wellness. In this review, health-specific technology designed specifically for the clinical setting includes health IT, EHRs, and telemedicine. We also included wellness informatics, defined as “a human-centered computing science focused on the design, deployment, and evaluation of human-facing technological solutions to promote and manage wellness acts such as the prevention of disease and the management of health” [30], in this review. Wellness informatics encompasses technology that may have little or no interaction with the health care system and is used primarily by the consumer [30]. For this review, wellness informatics tools included media technology created for other domains, such as television, radio, and computers.

### Search Strategy

From July to October 2011, we searched the online reference databases PubMed, Web of Knowledge, PsycINFO, and Engineering Village. For each database, we chose keywords to match the specific database’s thesaurus and used them to create search phrases. Each search phrase consisted of three key components: a word or phrase considering historically underserved populations, a word or phrase considering technology, and a phrase considering health, health access, or wellness (Table 1). Keywords about historically underserved populations included cultural diversity, ethnic groups, minority groups, cultural competency, ethnocentric, cross-cultural difference, racial and ethnic attitudes, racial and ethnic differences, and racial and ethnic discrimination. Keywords pertaining to health or health access included health education, patient acceptance of health care, attitudes to health, access to information, electronic health care, health, health system, and patient care. Finally, words considering technology included telemedicine, technology, medical technology, educational technology, medical information systems, eHealth, and health technology. When combined into the longer 3-part phrases, a total of 424 search phrases were used.

**Table 1:** Search terms by topic.

Historically underserved populations	Technology	Health and health access
Cultural diversity^a^	Telemedicine^a,b,c^	Health education^a^
Ethnic groups^a,d^	Technolog*^a,b,c^	Patient acceptance of healthcare/ethnology^a^
Medically underserved areas^a^	Medical technolog*^a,d^	Acceptance of healthcare^a^
Minority group^a^	Educational technology^a^	Attitudes to health^a^
Cross-sectional studies^a^	Electronic healthcare^d^	Access to information^a^
Cultural competenc*^a,d^	E-health^d^	Health knowledge, attitudes, practice^a^
Health status disparities^a^	Health technololog*^d^	Evaluation^d^
Disparit*^d^	Healthcare technolog*^d^	Health access^d^
Social factors^d ^	Medical information systems^b^	Technolog* acceptance^d^
Ethnocentric^d^	Medical computing^b^	Healthcare professionals^d^
Reference group culture^d^	Information technology^b,c,d^	Health system^d^
Cultur* bias^d^		Health^d^
Minorit*^d^		Healthcare^b^
Cultural aspects^b^		Patient care^b^
Culture bound syndromes^c^		Health disparities^c^
Ethnology^c^		Health attitudes^c^
Cross cultural differences^c^		Health knowledge^c^
Racial and ethnic attitudes^c^		Health impairments^c^
Racial and ethnic differences^c^		Health complaints^c^
Racial and ethnic groups^c^		
Race and ethnic discrimination^c^		

^a ^PubMed.

^b ^Engineering Village.

^c ^PsycINFO.

^d ^Web of Science.

### Inclusion and Exclusion Criteria

The scope of the review was focused by establishing inclusion and exclusion criteria. The selection criteria were that the paper (1) focused on a specific priority population(s), (2) discussed how the populations’ identity affected their experience within the health care system, and (3) discussed how technology use affected the experience.

We excluded studies if they (1) were published over 15 years ago (prior to 1996), (2) were not in English, (3) were conducted outside of the United States, (4) did not deal with health or wellness, (5) discussed mental health, end-of-life care, or dental care, or examined cost as the main variable, and (6) discussed the historically underserved population as a current or future employee of the health system instead of as a patient.

### Analysis

We used a methods-description approach to analyze papers that met the inclusion criteria. This method documented the objective characteristics (as they were described by the primary author) of each study’s methods [18]. In compliance with the methods-description approach and to ensure standardized data extraction of the reviewed papers, we created a data table [18] with the following sections: title, author, purpose, and key findings. After completing the table, we defined recurrent topics through coding. Coding is defined as the “analytical process through which concepts are identified and dimensions are discovered in data” [31]. Through use of coding, the following ideas were explored: the targeted historically underserved group, the health issue examined, how technology was used, evaluation techniques, and barriers to access or adoption (Multimedia Appendix 1 [4,9,12,13,15-17,32-149]).

## Results

**Figure 1:**
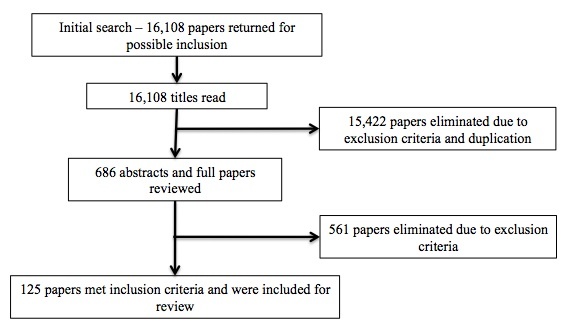
Flow diagram of the study selection process.

The 424 search phrases returned 16,108 papers. We excluded 15,422 papers as duplications or via the exclusion criteria through reading the titles. After reviewing the abstracts and full papers, we eliminated another 561papers as not meeting the inclusion criteria. A total of 125 papers met the inclusion criteria and were included in this review (Figure 1). An overview of the 125 papers can be found in Multimedia Appendix 1.

All selected papers discussed the health disparity facing the historically underserved group in question and the importance of closing the gap or reducing the disparity. One-quarter of the papers (32) focused only on the health disparity without analyzing a potential solution. The remaining 93 papers briefly mentioned the disparity but focused on accessing a possible solution to lessen the disparity. For instance, 1 report discussed the health disparities facing the Hispanic community in the background of the paper. However, the main purpose of the paper was to determine the effectiveness of *La Clinica del Pueblo, *a health education collaboration that uses radio to increase medical knowledge and have a positive affect on health behaviors [44]. The design and development of the technology was discussed in detail in only 13 of the reviewed papers. More often, the authors simply stated that technology was used in an attempt to lessen the disparity. Additionally, 5 papers were review papers; of the 5 review papers, 2 discussed diabetes [36,62], 2 discussed general health and health IT [9,15], and 1 discussed health literacy for people whose second language is English [65].

### Which Types of Technologies are Used to Address Negative Health Outcomes for Historically Underserved Populations?

We identified 30 types of technology in the selected papers (Table 2). The technology included both health informatics tools (personal digital assistant, radio, Internet, telephone, mobile computer, mobile phone, videotape, computer, kiosk, MP3, television, compact disc, multimedia tool, instant messaging, and fax machine) and more traditional health technology (general health IT, medical technology, telemedicine, telehealth, telemanagement, electronic medical records, personal health records, EHR, eHealth, assisted reproductive technology, high technology hospitals, rapid human immunodeficiency virus (HIV) testing, implantable cardioverter defibrillator, cochlear implants, and assistive technology). The technology was used in a variety of ways, including as educational tools, as pieces of interventions, or as collaboration tools between physicians and patients.

**Table 2:** Paper breakdown by technology.

Technology	Number of papers
Video	34
Internet (email, social networking sites)	23
Telemedicine	10
Computer (computers in clinics)	9
Television (advertisements and shows)	8
General health information technology	6
Electronic health record	6
Radio	5
Telephone	5
Mobile phone (text messaging)	5
Assisted reproductive technology	5
Multimedia tool	4
Assistive technology	4
Telehealth	3
Compact disc	2
Kiosk	2
Telemanagement	2
eHealth	2
Medical technology	1
Electronic medical record	1
Personal health record	1
Personal digital assistant	1
Mobile computer	1
High-technology hospitals	1
MP3	1
Rapid HIV^a ^testing	1
Implantable cardioverter defibrillator	1
Cochlear implants	1
Instant messaging (on a computer)	1
Fax machine	1

^a ^Human immunodeficiency virus.

### At Which Disease, Health Problem, or Potential Problem is the Technology Aimed?

The reviewed papers discussed 23 health issues (Table 3). Roughly one-quarter of the papers (33) did not focus on a single health topic, but instead discussed the general health of a population. Other papers examined health issues such as disease management (eg, diabetes, asthma, and obesity); health behaviors (eg, nutrition and smoking); and short-term issues (eg, breast-feeding, issues facing pregnant mothers, and child development).

Nearly half of the papers (62) examined the use of technology to create effective and culturally informed interventions (16 papers) or educational tools (46 papers). The reviewed papers pointed to many interventions and educational tools that were successfully designed for a historically underserved group. A study found that having famous athletes, musicians, and other celebrities from the African American community record commercials for adolescents’ MP3 players resulted in better health knowledge about asthma [95]. Another study created a telenovela for Latinas to discuss breast cancer in the dramatic and narrative format of a typical telenovela. Relating to the women on a cultural level, such as through the telenovela, resulted in higher breast cancer knowledge for the participants [144].

**Table 3:** Paper breakdown by health issue.

Health issue	Number of papers
General health	33
Cancer	17
Diabetes	14
HIV/AIDS^a^	14
Nutrition, physical activity	8
Sexually transmitted infections	7
Reproduction	5
Obesity	4
Cardiovascular disease, heart problems	4
Breast-feeding	3
Smoking	3
Asthma	3
Persons with disabilities	3
Pregnancy issues	2
Pharmacy	2
Sensorineural hearing loss	1
Organ donation	1
Hepatitis C	1
Health literacy	1
High blood pressure	1
Poison control	1
Hypertension	1
Child development	1

^a ^Human immunodeficiency virus/acquired immunodeficiency syndrome.

### Which Historically Underserved Groups are Technology-Based Interventions Designed for in the Literature?

The papers identified 19 different historically underserved populations (Table 4). Of the reviewed papers, 18 discussed multiple groups (eg, elderly African Americans or Hispanic women) and therefore appear in 2 categories in the table. In 8 the group in question self-identified as “racial and ethnic minorities.” We copied this term in this review paper only when the original author did not provide sufficient details to determine which racial or ethnic minorities were being examined. In addition to racial and ethnic minorities, the reviewed papers also included historically underserved groups that were characterized by their age, gender, location, and socioeconomic status.

**Table 4:** Paper breakdown by historically underserved group.

Historically underserved group	Number of papers
African American	64
Hispanic	51
Women (mothers)	26
Low socioeconomic status	11
Elderly	11
Adolescents, teens, and children	8
Racial and ethnic minorities	8
English as a second language	5
Native American and Alaskan	4
Men	4
Rural	4
Underresourced setting, underserved community	3
Community health center: underserved, low socioeconomic status, racial and ethnic groups	2
People getting tested for HIV^a^	2
Asian American	1
Immigrant	1
Homeless	1
People with AIDS^b^	1
People living with HIV	1

^a ^Human immunodeficiency virus.

^b ^Acquired immunodeficiency syndrome.

### How Are the Health Benefits and Technologies Evaluated?

Other than the 3 review papers, the papers all used formative technology evaluation. They used two forms of evaluation: (1) evaluation of health changes related to use of the technology and (2) evaluation of the technology itself; few papers (23) used both types of evaluation. A total of 76 of the papers evaluated health changes due to use of the technology (eg, changes in health knowledge, health behavior changes, biometric changes, or changes in health-related quality of life). Of the 107 papers that evaluated technology, 57 evaluated acceptance of the technology (satisfaction or acceptance, usefulness, and willingness to use), 14 evaluated usability (ease of use), 35 evaluated the user’s ability to access the technology (access or usage rates and number of websites or television advertisements with the desired information), and 1 measured improvements in technology literacy. In addition, 64 papers relied on the participants’ self-report to evaluate the technology, 14 measured ease of use, 22 measured usefulness of the technology, and 28 evaluated satisfaction with the technology. When an intervention or educational tool was evaluated, some of the authors (25 papers) measured improvement in participant health knowledge, while others measured behavior change (22 self-reported behavior changes and 18 observed behavior changes). Furthermore, 10 papers measured biometric changes in the observed health condition, 31 examined access and usage rates of the technology, and 7 recorded whether patients were interested in using the technology in the future. Finally, 4 papers measured the number of websites or television advertisements viewed by the population being studied. Table 5 lists the evaluation methods.

**Table 5:** Evaluation metrics.

Evaluation metric		Number of papers
**Evaluation of health changes related to use of the technology**		
	Health knowledge	25
	Behavior change (self-reported)	22
	Behavior change (observed)	18
	Biometric change	10
	Health-related quality of life	1
**Evaluation of the technology itself**		
	Access and usage rates	31
	Self-reported satisfaction and acceptance	28
	Usefulness (self-reported)	22
	Ease of use (self-reported)	14
	Willingness to use	7
	Number of websites or television advertisements with desired information	4
	Technology literacy improvement	1

## Discussion

The purpose of this study was to examine ways in which technology is being designed for historically underserved populations to facilitate or improve health care access and health outcomes. The reviewed studies focused on either (1) a defined historically underserved population, such as African Americans or people with a lower socioeconomic status, or (2) a historically underserved population, such as racial and ethnic minorities, as a group.

The results are organized into the four main questions. (1) Which types of technologies are used to address negative health outcomes for historically underserved populations? (2) At which disease, health problem, or potential problem is the technology aimed? (3) Which historically underserved groups are technology-based interventions designed for in the literature? (4) How are the health benefits and technologies evaluated?

### Which Types of Technologies are Used to Address Negative Health Outcomes for Historically Underserved Populations?

The papers discussed 30 different types of technology; half (15) are typically used within a clinical setting, while the remaining 15 types are often used outside of a medical setting. Technologies that are often used outside of a clinical setting were mentioned in the majority of papers (102 papers) and included technologies such as videotapes, Internet, computer, and radio. While not originally created for the health care system, these types of technology were readily adapted to aid health consumers. If a historically underserved population is already familiar with and has access to this type of technology, the technology might be an appropriate platform choice. For instance, 34 papers used videos to relay health messages. Videos are readily understood and easily accessed by the majority of the US population and therefore likely a good choice for health education or interventions aimed at historically underserved populations.

A total of 45 papers used technology typically used within a health care setting (eg, telemedicine, EHRs, or assisted reproductive technology). However, seven of these technologies (medical technology, electronic medical records, personal health records, high-technology hospitals, rapid HIV testing, implantable cardioverter defibrillator, and cochlear implants) were mentioned in only 1 paper [69,77,85,88,121,124,126]. Furthermore, telemedicine was the only type of health-specific technology mentioned in 10 or more papers.

Additionally, 16 papers discussed more than one type of technology, and the majority of these papers (14) mentioned two types of technology typically used outside of the medical office. The remaining 2 papers mentioned one type of each: one type of technology typically used at a clinic, and one type typically used outside of a clinic (telemedicine and videotapes [142], assistive technology and Internet [72]). None of the papers mentioned using more than one type of technology that is typically used inside a medical office.

Among the reviewed papers, videotapes were widely discussed as a method for interventions and educational tools (24 papers). Using videotapes instead of written materials to educate patients increased comprehension among breast cancer patients with low literacy skills [41]. Culturally tailored videotapes that employed characters of the same ethnic background as the patient influenced African American and Hispanic women on both a cognitive and emotional level [41]. Additionally, multiple studies showed increased trust among the patients when the narrator or main character of the educational videotape was the same ethnicity or race as the audience [47,144,150]. In addition, 2 studies demonstrated how storytelling can be used in videotapes to effectively communicate and educate patients about a specific health condition [78,122]. Videotapes were often complemented by other technology such as informational brochures [63], the radio [125], the computer [45,47], self-efficacy and skill-building exercises [53], multimedia tools [102,136], and telemedicine [142].

The Internet is highly used by health care professionals for interventions and education. One study showed the increased benefit of the Internet to individuals with lower incomes and education levels despite their lower use of the Internet to access health information [151]. Women, minorities, and poverty-stricken individuals (who are also part of the population with the fastest-growing rate of HIV/acquired immunodeficiency syndrome [AIDS]) are those most likely to not have access to the Internet [59]. While many historically underserved populations have lower access to the Internet on a computer, they have higher usage rates of mobile Internet access on handheld devices [151]. Almost two-thirds of African American (64%) and Hispanic people (63%) have wireless access to the Internet. In fact, more African American and Hispanic people own cell phones (87%) than their white counterparts (80%) and, further, these historically underserved populations use their phone data functions more than their white counterparts do [9]. Gibbons suggested that, due to the high usage rates, these tools could improve patient engagement and be an effective mode for interventions [9]. Crilly and colleagues suggested wireless handheld devices as a viable alternative for patients who face barriers due to geography [151]. Eyrich-Garg conducted a study on homeless individuals who faced barriers due to geography. Of the participants in his study, nearly half (44%) owned a mobile phone [59]. Of this 44%, one-fifth had accessed the Internet via their mobile phone in the past 30 days. For this reason, Eyrich-Garg suggested that health care providers could disseminate health information to the homeless through use of mobile phones [59].

Using mobile phones as a means to send information via text messaging is mentioned in the literature as a viable option for racial groups. Similar to their usage of mobile phones, African Americans use text messaging more than their white counterparts do [55]. Samal et al found that text messaging was an acceptable mode of information and communication technology for African American women in an urban sexually transmitted infection clinic [105]. Another study tested the feasibility of text messages as an HIV prevention method for young African American men. The results were positive and suggested that humor be used to initially engage the patient before providing an HIV fact later in the text [148]. While the research is new and applied to only a few select historically underserved populations, text messages are being used as a modality to disseminate health information to these populations.

To access the desired and undesired effects of technology and to search for relevant literature about a technology, a clear definition and delineation of technology is necessary [23]. When the technology is a surgical instrument or a piece of equipment, the definition is seldom a problem; however, other technologies are more complex and unformed, and require more thought to define (eg, wound care, fast-track surgery, or electronic medication). When a definition is created, the technology should be described from its material nature, its purpose, the degree of dissemination, and its maturity [23]. Kristensen and Sigmund suggested that the technology can be defined through a series of questions about how the technology is used for the disease or illness, or the technology [23]. To define the technologies in this review, we asked the following questions [23]. (1) Are there any special professional or technical requirements for operating the technology? (2) Are there factors that affect the application of the technology? (3) What is the purpose and application area of the technology? (4) At which disease, health problem, or potential problem is the technology aimed?

Questions 1 and 2 point to the need to effectively design technology that can overcome cultural differences that are exaggerated by the digital divide, health literacy, and language differences between historically underserved groups and the larger population. Every user needs to be able to operate and understand the technology to effectively access and use it to improve his or her health [23]. With regard to question 3, 23 identified 16 types of technology applications (Table 6). Question 4 is discussed in detail below.

**Table 6:** Types of technology

Application of technology	Number of papers
Intervention or education tool	62
Health management tool	19
Tool for communication with provider	6
Health record	5
Reproduction	5
Assistive technology	4
Information-gathering tool	3
Interpretation tool	2
Information and communication technology	1
Health information tool	1
Cardioverter defibrillation—medical technology	1
Cochlear implant—medical technology	1
Pharmacy tool	1
Drug advertisements	1
Knowledge acquisition	1
Health literacy assessment	1

### At Which Disease, Health Problem, or Potential Problem is the Technology Aimed?

Although 23 health issues were discussed in the reviewed papers, general health was discussed in one-quarter of the papers (33). The next five most mentioned health issues (cancer, diabetes, HIV/AIDS, nutrition and physical activity, and sexually transmitted infections) were mentioned in a disproportionate number of papers (60), while the remaining 17 health issues were mentioned in only 37 papers. Furthermore, eight of the health issues (sensorineural hearing loss, organ donation, hepatitis C, health literacy, high blood pressure, poison control, hypertension, and child development) were mentioned a only single paper each.

### Which Historically Underserved Groups are Technology-Based Interventions Designed for in the Literature?

The reviewed papers discussed 19 historically underserved groups. African American and Hispanic populations were mentioned at least twice as often (64 and 51 papers, respectively) as the second-largest target group (ie, women were mentioned in 26 papers). While African American and Hispanic populations were mentioned often, other racial and ethnic groups were rarely mentioned. Native Americans and Alaskan natives were mentioned in 4 papers and Asian Americans were mentioned in only 1 paper. The studies involving Native Americans and Alaskan natives provided an overview of the Indian Health Service [114] and evaluated the positive implementation of EHRs [115], a telehealth network [56], and library access through the Internet [146]. The studies demonstrated the importance of involving and empowering the community to successfully implement health IT [56,146]. More research is needed on this population to better understand the intricacies of implementing health IT in the Indian Health Service. Only 1 paper mentioned Asian Americans [126]; however, this paper was not singularly about Asian Americans. The paper showed that white and Asian American children were more likely to receive cochlear implants than their Hispanic and African American counterparts [126].

While the majority of the papers did not mention gender, when gender was mentioned, women were discussed in 26 papers, while men were specifically discussed in only 4 papers. Of the 26 papers focused on women, 16 described health issues specific to women (7 papers on reproduction, 7 on breast cancer, and 2 on breast-feeding). The remaining 10 papers discussed health conditions that are not gender specific and that could affect males (3 papers on HIV, 2 on obesity, and 1 on the remaining health issues: general health, cardiovascular disease, sexually transmitted infections, nutrition, and cancer). Of the 4 papers dedicated to men, 1 discussed prostate cancer, which is specific only to men; however, the remaining 3 papers discussed HIV and sexually transmitted infections, which can also affect women. While it is understandable that papers discussing gender-specific health issues such as breast cancer or prostate cancer would focus on a single gender, 10 papers targeted only women and 3 papers targeted only men while addressing a non-gender-specific health issue.

It is important to note that attitudes toward technological interventions vary between historically underserved populations, not just between majority populations and historically underserved populations. A single intervention will not necessarily work for two separate racial or ethnic groups; interventions should be tailored to each population to be most effective. For instance, 1 study found that African American and Hispanic populations have different concerns regarding telemedicine [12]. While African American participants were concerned by the physical absence of the health care professional and the ability to monitor their qualifications, Hispanic participants were concerned with whether telemedicine would be available to uninsured or undocumented individuals.

### How are the Health Benefits and Technologies Evaluated?

The review papers used 12 types of evaluation. While we expected that most of the papers would use quantitative evaluation techniques, only half of the papers used these techniques. Objective evaluations were used in 90 papers (31 papers measured access or usage rates, 25 measured changes in health knowledge, 18 measured behavior changes, 10 measured biometric changes, 4 counted the number of websites or television advertisements with the desired information, 1 measured improvements in technology literacy, and 1 measured health-related quality of life). Self-reported measures were used in 93 papers (28 papers measured self-reported satisfaction or acceptance, 22 measured self-reported behavior changes, 22 measured self-reported usefulness, 14 measured self-reported ease of use, and 7 measure willingness to use the technology). Though 10 of the papers measured biometric changes, most of the papers did not evaluate the effects of the technology on health outcomes. Instead, the papers evaluated intermediate measures such as behavior changes or access rates of the technology.

Of the 67 papers that tested a culturally informed technology, 66 found the technology successful in at least one of the evaluated metrics; this points to the fact that health technology is an effective method to improve the health of historically underserved populations. The one study that did not have success aimed to improve HIV risk and sexual behaviors through a culturally appropriate educational video for 15- to 19-year-old black males [51]. Instead, the researchers suggested that an African American health educator conduct face-to-face interventions in order to have a greater impact.

### Conclusion

This review illustrates how technology is being used by historically underserved populations to facilitate or improve their health care access and health and wellness outcomes. Synthesis of the literature points to the benefit of accounting for the end user’s culture when designing health technology. A person’s culture shapes how health information is received, what a health consumer considers a health problem, how symptoms are expressed, who should provide treatment, and what treatment should be provided [152]. The review conveys that culturally informed technology affects the health outcomes of the historically underserved populations facing health disparities in the United States.

#### Which Types of Technologies are Used to Address Negative Health Outcomes for Historically Underserved Populations?

The reviewed papers discussed 30 different types of technology, both those typically used inside a medical setting and those typically used outside of a medical setting. Health IT can lessen barriers facing historically underserved populations [11]. However, administrators and physicians should carefully analyze the type of technology they choose to implement, as different types of technology are better than others at overcoming certain barriers. Since different historically underserved populations face distinct barriers, choosing a technology type should be an informed decision. For instance, people living in rural and underresourced areas face extra barriers related to provider availability and transportation [153]. The use of telemedicine, where the providers can be located in a different region, can overcome these barriers and aid historically underserved populations in accessing patient-centered care [4,37]. However, of the reviewed papers, only 4 discussed using telemedicine to aid underresourced or rural populations [4,12,37,35]. In another example, using culturally tailored technology that places little financial burden on the consumer and is easy to use, such as videotapes, television advertisements, and compact discs, can help mitigate health disparities facing individuals with low socioeconomic status [154]. This review provides evidence that these technologies have been implemented to help historically underserved populations (34 video papers, 8 television papers, 2 compact disc papers). Additionally, the type of technology with the greatest potential to aid individuals facing multiple chronic conditions is EHRs [155]. However, none of the 32 chronic disease papers (diabetes, HIV/AIDS, asthma, and hypertension) used EHRs.

Choosing an appropriate type of technology is not enough; the technology should be tailored toward the intended population, as personally relevant health technology is more likely than more broad technology to change behavior [1].

#### At Which Disease, Health Problem, or Potential Problem is the Technology Aimed?

The reviewed papers discussed 23 health issues, with 33 of the papers discussing general health concerns. Since the US federal government requires recipients of federal funds to provide language assistance services, including bilingual staff and interpreters, at no cost to the patient [152], it is surprising that health literacy was not mentioned in more papers. Technology can easily translate difficult health terms and issues into more easily understood concepts for laypeople. Recent reports by the Institute of Medicine and Agency for Healthcare Research and Quality (AHRQ) recommended that future research examine culture and cultural differences when measuring health literacy [156]. Specifically, the 2004 AHRQ report recommended that covariates such as socioeconomic status or education level should be further explored [157]. In addition, it is surprising that chronic diseases were not mentioned in more papers, as chronic diseases are the leading cause of health disparities [36]. Technology can help health consumers manage their overall health behaviors and medicine intake, and thus we expected that more papers would have discussed chronic diseases.

Nearly half of the papers discussed how the technology was used to create culturally informed interventions or educational tools. Obtaining access to culturally appropriate and accessible health education is a necessary piece of receiving high-quality, patient-centered care [154]. Similarly, the reviewed papers support Barrera et al’s findings that culturally appropriate health interventions are more effective than usual care. However, there are important limitations to previous research [158]. Since culturally adapted interventions are seldom directly compared with nonculturally informed interventions, it is difficult to state with certainty that the cultural aspect of the intervention was an important piece of the success of the intervention.

#### Which Historically Underserved Groups are Technology-Based Interventions Designed for in the Literature?

The papers included in this review highlight a relatively limited number of historically underserved groups (19). However, the review papers did discuss seven priority populations defined by the AHRQ. The AHRQ focused on seven priority populations as specified by Congress in the Healthcare Research and Quality Act of 1999: racial and ethnic minorities, low-income groups, women, children, older adults, residents of rural areas, and individuals with special health care needs, such as individuals with disabilities and individuals who need chronic care or end-of-life care [153]. According to AHRQ, racial minority groups are white people, black people, Asians, Native Hawaiian or other Pacific Islanders, American Indian and Alaska natives, and people who belong to more than one race; ethnic minority groups are Hispanic or Latino [153]. Within the reviewed papers, chronic care and disabilities were not discussed as characterizing historically underserved groups but were mentioned as health issues facing the different populations. In addition to the AHRQ priority populations, the reviewed papers discussed an additional four historically underserved groups: people who speak English as a second language, men, immigrants, and homeless people.

Of the 19 historically underserved groups discussed in the reviewed papers, 11 of these groups were discussed in fewer than 5 papers. Further, five groups (Asian Americans, immigrants, the homeless, people with AIDS, and people living with HIV) were discussed in a single paper. The discrepancy in the number of papers reviewed per historically underserved population is potentially problematic, as it can result in gaps in information regarding the less-studied populations [153]. Furthermore, understudied populations are left out of relevant discourse and in turn rendered invisible and powerless [159]. It is important to study all historically underserved groups to avoid this invisibility and bring awareness to the populations.

The reviewed papers tended to examine one identity that an individual might hold. In addition to studying historically underserved groups separately, researchers should examine populations from an intersectional theoretical perspective. Intersectionality refers to particular forms of intersecting oppressions [160] such as being both Hispanic and an older individual. The combination of these two identities creates different barriers for the patient than either single identity would create on its own. While the papers discussed 18 combinations of cultural groups, they did not adequately theorize the issue of intersectionality and therefore cannot fully understand the barriers and problems facing individuals within the group.

#### How are the Health Benefits and Technologies Evaluated?

We identified 2 main forms of evaluation in the reviewed papers: evaluation of health changes related to use of the technology and evaluation of the technology itself. A fraction of the papers (23) used both types of evaluation. A wide range of evaluation metrics were used; about half of the papers (64) used self-reported measures as part of their evaluation, while 10 papers measured biometric changes. Even though previous research found self-reports to not be an accurate predictor of health information competencies [161], 32 papers used only self-reported metrics.

The reviewed papers did not include a validated method to evaluate the specific cultural aspect of the health technology. Design processes should be reported in the research so that best practices can be created for culturally relevant design methods. Only 13 of the reviewed papers provided detail on the design process of the interventions and educational tools. Future research should evaluate metrics for culturally informed health technology. These metrics will need to be adapted and changed for different cultural groups, as diverse cultural groups expect different criteria from their health technology.

### Study Limitations

We followed systematic review methodology; however, this method has several limitations. Systematic reviews can only assess published work and report on the findings in those articles. Other potential limitations include the use of a single reviewer and the exclusion of studies regarding mental health, end-of-life care, and dental care.

### Future Recommendations

More research about culturally informed technology for health is needed. In conjunction with this research, it is imperative for researchers to continue collecting data on cultural populations [162]. Gaps in knowledge about the access to and use of health services by historically underserved populations exist in terms of learning practices, methods to navigate services, and help-seeking behaviors [163]. Further research is necessary to understand the limitations of the data and avoid overgeneralizations [162]. Future recommendations include the following:

- Theoretical models and perspectives are needed to design culturally informed technologies.
- Methodologically, more research should be conducted to create a culturally informed approach to the design of health technology geared toward historically underserved populations. While methods should vary based on the technology, cultural population, and health issue, a broad methodology should be recommended for the future design of culturally informed health technology. This methodology might include formative research, which can aid researchers in overcoming their own implicit biases by using participatory methods to help them understand the population, create programs specific to the population’s needs, and ensure the programs are acceptable to the population through pilot testing [164]. Formative research includes qualitative research methods such as focus groups, interviews with key informants, surveys, and field notes. When using formative research to develop culturally informed health IT, key informants might include cultural theorists.
- Financial incentives should be provided to organizations that adopt technology for historically underserved populations. The financial burden of purchasing, implementing, and maintaining health IT serves as a barrier to the adoption among underresourced providers who frequently serve lower socioeconomic patients [14].

Recommendations related to the type of technology chosen are as follows:

- When designing or implementing health technology for historically underserved populations, the type of technology should be carefully considered. Barriers to access and use of health IT differ between populations; different types of technology can be used to overcome distinct barriers. Therefore, the choice of technology type is important. Future research should create a comprehensive list of which types of technology would be most beneficial for each group. For instance, telemedicine is a useful tool to reach rural populations, and mobile telephones are a useful tool to reach African American populations.
- Trust and lack of cultural relevance have been found to be a barrier, as lack of trust in the technology, technical problems, or confusing instructions have a negative impact on adoption and usage rates among historically underserved populations [9]. More research is necessary to determine whether patients’ culture changes their level of trust in culturally informed health IT.
- Future studies should examine how to best diffuse technology into a population [165]. When implementing an intervention, researchers should evaluate the readiness of the intended population.
- As new technology is invented and as the cost of current technology decreases, culturally informed health technology should be adapted. For instance, social media, which have rapidly grown in the 21st century [166], should be further examined as a possible method to reach historically underserved populations. Social media have already started to enter the health care system through online patient communities such as PatientsLikeMe, QuitNet, and CureTogether. These networks create spaces for patients to discuss specific conditions and share their experiences. If access does not serve as a barrier, research shows that these social networks can be useful for historically underserved populations [9]. Social media may prove to be a cheaper way to access geographically isolated populations.

Recommendations related to the disease, health problem, or potential problem are the following:

- Future studies should use culturally informed health IT for chronic disease management. The emphasis on self-management support programs has shifted from pedagogical education with education content defined by health care professionals to an individualized approach that addresses the specific needs of a patient’s situation [167]. Future research should examine how to best use technology to aid in the disease management of historically underserved populations with chronic diseases.

The recommendation related to the evaluation of the technology is the following:

- The evaluation process should be standardized to create a benchmark for culturally informed health IT. Participatory approaches should be used when possible to evaluate technologies, but metrics related to culturally informed design are needed. While research should dictate these metrics, possible metrics might include issues surrounding access, usability, perceived usefulness, and cultural appropriateness.

## Multimedia Appendix 1

Overview of reviewed papers.

## Abbreviations

AHRQ: Agency for Healthcare Research and Quality
AIDS: acquired immunodeficiency syndrome
EHR: electronic health record
HIV: human immunodeficiency virus
IT: information technology

## References

[1] Gibbons MC (2005). A historical overview of health disparities and the potential of eHealth solutions. J Med Internet Res.

[2] Pecukonis EV, Cornelius L, Parrish M (2003). The future of health social work. Soc Work Health Care.

[3] Partida Y (2007). Addressing language barriers: building response capacity for a changing nation. J Gen Intern Med.

[4] Coen Buckwalter K, Lindsey Davis L, Wakefield BJ, Kienzle MG, Ann Murray M (2002). Telehealth for elders and their caregivers in rural communities. Fam Community Health.

[5] Carter-Pokras O, Baquet C (2002). What is a "health disparity"?. Public Health Rep.

[6] Betancourt JR, Green AR, Carrillo JE, Ananeh-Firempong O (2003). Defining cultural competence: a practical framework for addressing racial/ethnic disparities in health and health care. Public Health Rep.

[7] Baquet CR, Carter-Pokras O, Bengen-Seltzer B (2004). Healthcare disparities and models for change. Am J Manag Care.

[8] (2011). US Centers for Disease Control and Prevention, National Center for Health Statistics.

[9] Christopher Gibbons M (2011). Use of health information technology among racial and ethnic underserved communities. Perspect Health Inf Manag.

[10] (2009). World Health Organization.

[11] Leavitt MO (2011). Health Information Technology Initiative: Major Accomplishments: 2004-2006.

[12] George SM, Hamilton A, Baker R (2009). Pre-experience perceptions about telemedicine among African Americans and Latinos in South Central Los Angeles. Telemed J E Health.

[13] Fiscella K, Geiger HJ (2006). Health information technology and quality improvement for community health centers. Health Aff (Millwood).

[14] Blumenthal D, DesRoches C, Foubister V (2008). George Washington University, Massachusetts General Hospital, Robert Wood Johnson Foundation.

[15] Millery M, Kukafka R (2010). Health information technology and quality of health care: strategies for reducing disparities in underresourced settings. Med Care Res Rev.

[16] Li C, West-Strum D (2010). Patient panel of underserved populations and adoption of electronic medical record systems by office-based physicians. Health Serv Res.

[17] Custodio R, Gard AM, Graham G (2009). Health information technology: addressing health disparity by improving quality, increasing access, and developing workforce. J Health Care Poor Underserved.

[18] Cooper H (1998). Synthesizing Research: A Guide for Literature Reviews. 3rd edition.

[19] Paniagua FA (2005). Assessing and Treating Culturally Diverse Clients: A Practical Guide. 3rd edition.

[20] Eysenbach G (2001). What is e-health?. J Med Internet Res.

[21] Larson JS (1999). The conceptualization of health. Med Care Res Rev.

[22] Varshney U (2007). Pervasive healthcare and wireless health monitoring. Mobile Netw Appl.

[23] Kristensen F, Sigmund H (2007). Health Technology Assessment Handbook. 2nd edition.

[24] Thompson TG, Brailer DJ (2004). The Decade of Health Information Technology: Delivering Consumer-Centric and Information-Rich Health Care.

[25] Or CK, Karsh BT (2009). A systematic review of patient acceptance of consumer health information technology. J Am Med Inform Assoc.

[26] Magro A, Swarz J, Ousley A (2010). CancerSPACE: an interactive e-learning tool aimed to improve cancer screening rates. J Comput Mediat Commun.

[27] (2006). World Health Organization Western Pacific Region.

[28] Garets D, Davis M (2005). Healthcare Informatics Online.

[29] (2006). Markle Foundation.

[30] Grinter RE, Siek KA, Grimes A (2010). Is wellness informatics a field of human-centered health informatics?. interactions.

[31] Van De Belt TH, Engelen LJ, Berben SA, Schoonhoven L (2010). Definition of Health 2.0 and Medicine 2.0: a systematic review. J Med Internet Res.

[32] Abbatangelo-Gray J, Byrd-Bredbenner C, Austin SB (2008). Health and nutrient content claims in food advertisements on Hispanic and mainstream prime-time television. J Nutr Educ Behav.

[33] Alvaro EM, Siegel JT, Crano WD, Dominick A (2010). A mass mediated intervention on Hispanic live kidney donation. J Health Commun.

[34] Anderson RM, Funnell MM, Arnold MS, Barr PA, Edwards GJ, Fitzgerald JT (2000). Assessing the cultural relevance of an education program for urban African Americans with diabetes. Diabetes Educ.

[35] Arora S, Thornton K, Jenkusky SM, Parish B, Scaletti JV (2007). Project ECHO: linking university specialists with rural and prison-based clinicians to improve care for people with chronic hepatitis C in New Mexico. Public Health Rep.

[36] Baig AA, Wilkes AE, Davis AM, Peek ME, Huang ES, Bell DS, Chin MH (2010). The use of quality improvement and health information technology approaches to improve diabetes outcomes in African American and Hispanic patients. Med Care Res Rev.

[37] Balamurugan A, Hall-Barrow J, Blevins MA, Brech D, Phillips M, Holley E, Bittle K (2009). A pilot study of diabetes education via telemedicine in a rural underserved community--opportunities and challenges: a continuous quality improvement process. Diabetes Educ.

[38] Bell DS, Daly DM, Robinson P (2003). Is there a digital divide among physicians? A geographic analysis of information technology in Southern California physician offices. J Am Med Inform Assoc.

[39] Bertera EM, Tran BQ, Wuertz EM, Bonner A (2007). A study of the receptivity to telecare technology in a community-based elderly minority population. J Telemed Telecare.

[40] Black MM, Teti LO (1997). Promoting mealtime communication between adolescent mothers and their infants through videotape. Pediatrics.

[41] Borrayo EA (2004). Where's Maria? A video to increase awareness about breast cancer and mammography screening among low-literacy Latinas. Prev Med.

[42] Buchanan LM, Khazanchi D (2010). A PDA intervention to sustain smoking cessation in clients with socioeconomic vulnerability. West J Nurs Res.

[43] Calderon Y, Leider J, Hailpern S, Haughey M, Ghosh R, Lombardi P, Bijur P, Bauman L (2009). A randomized control trial evaluating the educational effectiveness of a rapid HIV posttest counseling video. Sex Transm Dis.

[44] Calles-Escandón J, Hunter JC, Langdon SE, Gómez EM, Duren-Winfield VT, Woods KF (2009). La Clínica del Pueblo: a model of collaboration between a private media broadcasting corporation and an academic medical center for health education for North Carolina Latinos. J Immigr Minor Health.

[45] Campbell MK, Honess-Morreale L, Farrell D, Carbone E, Brasure M (1999). A tailored multimedia nutrition education pilot program for low-income women receiving food assistance. Health Educ Res.

[46] Carter EL, Nunlee-Bland G, Callender C (2011). A patient-centric, provider-assisted diabetes telehealth self-management intervention for urban minorities. Perspect Health Inf Manag.

[47] Champion VL, Springston JK, Zollinger TW, Saywell RM, Monahan PO, Zhao Q, Russell KM (2006). Comparison of three interventions to increase mammography screening in low income African American women. Cancer Detect Prev.

[48] Chan YF, Alagappan K, Rella J, Bentley S, Soto-Greene M, Martin M (2010). Interpreter services in emergency medicine. J Emerg Med.

[49] Choi N (2011). Relationship between health service use and health information technology use among older adults: analysis of the US National Health Interview Survey. J Med Internet Res.

[50] Clifton GD, Byer H, Heaton K, Haberman DJ, Gill H (2003). Provision of pharmacy services to underserved populations via remote dispensing and two-way videoconferencing. Am J Health Syst Pharm.

[51] DeLamater J, Wagstaff DA, Havens KK (2000). The impact of a culturally appropriate STD/AIDS education intervention on black male adolescents' sexual and condom use behavior. Health Educ Behav.

[52] Delorme DE, Huh J, Reid LN (2010). Evaluation, use, and usefulness of prescription drug information sources among Anglo and Hispanic Americans. J Health Commun.

[53] DeMarco RF, Kendricks M, Dolmo Y, Looby SE, Rinne K (2009). The effect of prevention messages and self-efficacy skill building with inner-city women at risk for HIV infection. J Assoc Nurses AIDS Care.

[54] DeMarco R, Norris AE (2004). Culturally relevant HIV interventions: transcending ethnicity. J Cult Divers.

[55] Denizard-Thompson NM, Feiereisel KB, Stevens SF, Miller DP, Wofford JL (2011). The digital divide at an urban community health center: implications for quality improvement and health care access. J Community Health.

[56] Doorenbos AZ, Demiris G, Towle C, Kundu A, Revels L, Colven R, Norris TE, Buchwald D (2011). Developing the Native People for Cancer Control Telehealth Network. Telemed J E Health.

[57] Eddens KS, Kreuter MW, Morgan JC, Beatty KE, Jasim SA, Garibay L, Tao D, Buskirk TD, Jupka KA (2009). Disparities by race and ethnicity in cancer survivor stories available on the web. J Med Internet Res.

[58] Essien EJ, Meshack AF, Peters RJ, Ogungbade GO, Osemene NI (2005). Strategies to prevent HIV transmission among heterosexual African-American men. BMC Public Health.

[59] Eyrich-Garg KM (2010). Mobile phone technology: a new paradigm for the prevention, treatment, and research of the non-sheltered "street" homeless?. J Urban Health.

[60] Feinberg EC, Larsen FW, Catherino WH, Zhang J, Armstrong AY (2006). Comparison of assisted reproductive technology utilization and outcomes between Caucasian and African American patients in an equal-access-to-care setting. Fertil Steril.

[61] Finkelstein J, Cha E, Dennison CR (2010). Exploring feasibility of home telemanagement in African Americans with congestive heart failure. Stud Health Technol Inform.

[62] Fitzner K, Dietz DA, Moy E (2011). How innovative treatment models and data use are improving diabetes care among older African American adults. Popul Health Manag.

[63] Frosch DL, Légaré F, Mangione CM (2008). Using decision aids in community-based primary care: a theory-driven evaluation with ethnically diverse patients. Patient Educ Couns.

[64] Gans KM, Kumanyika SK, Lovell HJ, Risica PM, Goldman R, Odoms-Young A, Strolla LO, Decaille DO, Caron C, Lasater TM (2003). The development of SisterTalk: a cable TV-delivered weight control program for black women. Prev Med.

[65] Garcia-Castillo D, Fetters MD (2007). Quality in medical translations: a review. J Health Care Poor Underserved.

[66] Gilbert MR, Masucci M, Homko C, Bove AA (2008). Theorizing the digital divide: information and communication technology use frameworks among poor women using a telemedicine system. Geoforum.

[67] Gordon NP, Iribarren C (2008). Health-related characteristics and preferred methods of receiving health education according to dominant language among Latinos aged 25 to 64 in a large Northern California health plan. BMC Public Health.

[68] Grindel CG, Brown L, Caplan L, Blumenthal D (2004). The effect of breast cancer screening messages on knowledge, attitudes, perceived risk, and mammography screening of African American women in the rural South. Oncol Nurs Forum.

[69] Groeneveld PW, Sonnad SS, Lee AK, Asch DA, Shea JE (2006). Racial differences in attitudes toward innovative medical technology. J Gen Intern Med.

[70] Gross SM, Caulfield LE, Bentley ME, Bronner Y, Kessler L, Jensen J, Paige VM (1998). Counseling and motivational videotapes increase duration of breast-feeding in African-American WIC participants who initiate breast-feeding. J Am Diet Assoc.

[71] Guzman A (2008). Learning about assistive technology: Hispanics and a national sample. Assist Technol.

[72] Guzman A, Ostrander N (2009). Hispanics' awareness of assistive technology. Assist Technol.

[73] Helft PR, Eckles RE, Johnson-Calley CS, Daugherty CK (2005). Use of the internet to obtain cancer information among cancer patients at an urban county hospital. J Clin Oncol.

[74] Helion AM, Reddy DM, Kies AL, Morris DR, Wilson CM (2008). Influence of communicator's race on efficacy of an HIV/STD prevention intervention among African American and Caucasian college students. Public Health Nurs.

[75] Henderson VR, Kelly B (2005). Food advertising in the age of obesity: content analysis of food advertising on general market and african american television. J Nutr Educ Behav.

[76] Hoffman AM, Redding CA, Goldberg D, Añel D, Prochaska JO, Meyer PM, Pandey D (2006). Computer expert systems for African-American smokers in physicians offices: a feasibility study. Prev Med.

[77] Horan TA, Botts NE, Burkhard RJ (2010). A multidimensional view of personal health systems for underserved populations. J Med Internet Res.

[78] Houston TK, Allison JJ, Sussman M, Horn W, Holt CL, Trobaugh J, Salas M, Pisu M, Cuffee YL, Larkin D, Person SD, Barton B, Kiefe CI, Hullett S (2011). Culturally appropriate storytelling to improve blood pressure: a randomized trial. Ann Intern Med.

[79] Jantz C, Anderson J, Gould SM (2002). Using computer-based assessments to evaluate interactive multimedia nutrition education among low-income predominantly Hispanic participants. J Nutr Educ Behav.

[80] Jha AK, Bates DW, Jenter C, Orav EJ, Zheng J, Cleary P, Simon SR (2009). Electronic health records: use, barriers and satisfaction among physicians who care for black and Hispanic patients. J Eval Clin Pract.

[81] Kalichman SC, Cherry C, Browne-Sperling F (1999). Effectiveness of a video-based motivational skills-building HIV risk-reduction intervention for inner-city African American men. J Consult Clin Psychol.

[82] Kalichman SC, Weinhardt L, Benotsch E, Cherry C (2002). Closing the digital divide in HIV/AIDS care: development of a theory-based intervention to increase Internet access. AIDS Care.

[83] Kaye HS, Yeager P, Reed M (2008). Disparities in usage of assistive technology among people with disabilities. Assist Technol.

[84] Kelly NR, Huffman LC, Mendoza FS, Robinson TN (2003). Effects of a videotape to increase use of poison control centers by low-income and Spanish-speaking families: a randomized, controlled trial. Pediatrics.

[85] Kim TH, Samson LF, Lu N (2010). Racial/ethnic disparities in the utilization of high-technology hospitals. J Natl Med Assoc.

[86] Kreuter MW, Holmes K, Alcaraz K, Kalesan B, Rath S, Richert M, McQueen A, Caito N, Robinson L, Clark EM (2010). Comparing narrative and informational videos to increase mammography in low-income African American women. Patient Educ Couns.

[87] Leeman-Castillo B, Beaty B, Raghunath S, Steiner J, Bull S (2010). LUCHAR: using computer technology to battle heart disease among Latinos. Am J Public Health.

[88] Li J, Garcia S, Castro HK, DeForge BR, Hise MK, Finkelstein J (2007). Acceptance and expectations of information technology to support hypertension self-care in African Americans: a qualitative inquiry. AMIA Annu Symp Proc.

[89] Lorence DP, Park H, Fox S (2006). Racial disparities in health information access: resilience of the Digital Divide. J Med Syst.

[90] Mandl KD, Katz SB, Kohane IS (1998). Social equity and access to the World Wide Web and E-mail: implications for design and implementation of medical applications. Proc AMIA Symp.

[91] Mackert M, Kahlor L, Tyler D, Gustafson J (2009). Designing e-health interventions for low-health-literate culturally diverse parents: addressing the obesity epidemic. Telemed J E Health.

[92] McCarthy-Keith DM, Schisterman EF, Robinson RD, O'Leary K, Lucidi RS, Armstrong AY (2010). Will decreasing assisted reproduction technology costs improve utilization and outcomes among minority women?. Fertil Steril.

[93] Mendelsohn AL, Valdez PT, Flynn V, Foley GM, Berkule SB, Tomopoulos S, Fierman AH, Tineo W, Dreyer BP (2007). Use of videotaped interactions during pediatric well-child care: impact at 33 months on parenting and on child development. J Dev Behav Pediatr.

[94] Miller EA, West DM, Wasserman M (2007). Health information Websites: characteristics of US users by race and ethnicity. J Telemed Telecare.

[95] Mosnaim GS, Cohen MS, Rhoads CH, Rittner SS, Powell LH (2008). Use of MP3 players to increase asthma knowledge in inner-city African-American adolescents. Int J Behav Med.

[96] Outley CW, Taddese A (2006). A content analysis of health and physical activity messages marketed to African American children during after-school television programming. Arch Pediatr Adolesc Med.

[97] Nicklas TA, Goh ET, Goodell LS, Acuff DS, Reiher R, Buday R, Ottenbacher A (2011). Impact of commercials on food preferences of low-income, minority preschoolers. J Nutr Educ Behav.

[98] Noell J, Ary D, Duncan T (1997). Development and evaluation of a sexual decision-making and social skills program: "the choice is yours--preventing HIV/STDs". Health Educ Behav.

[99] Olshefsky AM, Zive MM, Scolari R, Zuñiga M (2007). Promoting HIV risk awareness and testing in Latinos living on the U.S.-Mexico border: the Tú No Me Conoces social marketing campaign. AIDS Educ Prev.

[100] Pérez-Escamilla R, Himmelgreen D, Bonello H, Peng YK, Mengual G, González A, Méndez I, Cruz J, Phillips LM (2000). Marketing nutrition among urban Latinos: the SALUD! campaign. J Am Diet Assoc.

[101] Powe BD, Weinrich S (1999). An intervention to decrease cancer fatalism among rural elders. Oncol Nurs Forum.

[102] Prokhorov AV, Kelder SH, Shegog R, Murray N, Peters R, Agurcia-Parker C, Cinciripini PM, de Moor C, Conroy JL, Hudmon KS, Ford KH, Marani S (2008). Impact of A Smoking Prevention Interactive Experience (ASPIRE), an interactive, multimedia smoking prevention and cessation curriculum for culturally diverse high-school students. Nicotine Tob Res.

[103] Ralph LJ, Berglas NF, Schwartz SL, Brindis CD (2011). Finding teens in TheirSpace: using social networking sites to connect youth to sexual health services. Sex Res Social Policy.

[104] Riegel B, Carlson B, Glaser D, Romero T (2006). Randomized controlled trial of telephone case management in Hispanics of Mexican origin with heart failure. J Card Fail.

[105] Samal L, Hutton HE, Erbelding EJ, Brandon ES, Finkelstein J, Chander G (2010). Digital divide: variation in internet and cellular phone use among women attending an urban sexually transmitted infections clinic. J Urban Health.

[106] Sánchez JP, Kaltwassar S, McClellan M, Burton WB, Blank A, Calderon Y (2010). Educational video tool to increase syphilis knowledge among black and Hispanic male patients. J Health Care Poor Underserved.

[107] Sánchez JP, Guilliames C, Sánchez NF, Calderon Y, Burton WB (2010). Video tool to promote knowledge of syphilis among black and Hispanic men recruited from clinical and non-clinical settings. J Community Health.

[108] Scheinmann R, Chiasson MA, Hartel D, Rosenberg TJ (2010). Evaluating a bilingual video to improve infant feeding knowledge and behavior among immigrant Latina mothers. J Community Health.

[109] Schillinger D, Hammer H, Wang F, Palacios J, McLean I, Tang A, Youmans S, Handley M (2008). Seeing in 3-D: examining the reach of diabetes self-management support strategies in a public health care system. Health Educ Behav.

[110] Schitai A (2004). Caring for Hispanic patients interactively: simulations and practices for allied health professionals. J Nurses Staff Dev.

[111] Schnall R, Gordon P, Camhi E, Bakken S (2011). Perceptions of factors influencing use of an electronic record for case management of persons living with HIV. AIDS Care.

[112] Seifer DB, Frazier LM, Grainger DA (2008). Disparity in assisted reproductive technologies outcomes in black women compared with white women. Fertil Steril.

[113] Seifer DB, Zackula R, Grainger DA, Society for Assisted Reproductive Technology Writing Group Report (2010). Trends of racial disparities in assisted reproductive technology outcomes in black women compared with white women: Society for Assisted Reproductive Technology 1999 and 2000 vs. 2004-2006. Fertil Steril.

[114] Sequist TD, Cullen T, Ayanian JZ (2005). Information technology as a tool to improve the quality of American Indian health care. Am J Public Health.

[115] Sequist TD, Cullen T, Hays H, Taualii MM, Simon SR, Bates DW (2007). Implementation and use of an electronic health record within the Indian Health Service. J Am Med Inform Assoc.

[116] Serou A, Quintero R (2011). Media influence on awareness and utilization of assisted reproduction technology in Hispanic populations. Fertil Steril.

[117] Shea S, IDEATel Consortium (2007). The Informatics for Diabetes and Education Telemedicine (IDEATel) project. Trans Am Clin Climatol Assoc.

[118] Shea S, Starren J, Weinstock RS, Knudson PE, Teresi J, Holmes D, Palmas W, Field L, Goland R, Tuck C, Hripcsak G, Capps L, Liss D (2002). Columbia University's Informatics for Diabetes Education and Telemedicine (IDEATel) Project: rationale and design. J Am Med Inform Assoc.

[119] Shields AE, Shin P, Leu MG, Levy DE, Betancourt RM, Hawkins D, Proser M (2007). Adoption of health information technology in community health centers: results of a national survey. Health Aff (Millwood).

[120] Singh H, Fox SA, Petersen NJ, Shethia A, Street RL (2009). Older patients' enthusiasm to use electronic mail to communicate with their physicians: cross-sectional survey. J Med Internet Res.

[121] Smith LV, Rudy ET, Javanbakht M, Uniyal A, Sy LS, Horton T, Kerndt PR (2006). Client satisfaction with rapid HIV testing: comparison between an urban sexually transmitted disease clinic and a community-based testing center. AIDS Patient Care STDS.

[122] Sobel RM, Paasche-Orlow MK, Waite KR, Rittner SS, Wilson EA, Wolf MS (2009). Asthma 1-2-3: a low literacy multimedia tool to educate African American adults about asthma. J Community Health.

[123] Sorensen L, Gavier M, Hellesø R (2009). Latina breast cancer survivors informational needs: information partners. Stud Health Technol Inform.

[124] Stanley A, DeLia D, Cantor JC (2007). Racial disparity and technology diffusion: the case of cardioverter defibrillator implants, 1996-2001. J Natl Med Assoc.

[125] Stelger G, Samkoff J, Karoullas J (2003). A program of interventions designed to increase mammography rates in women ages 50 years and older for an underserved racial minority. J Health Hum Serv Adm.

[126] Stern RE, Yueh B, Lewis C, Norton S, Sie KC (2005). Recent epidemiology of pediatric cochlear implantation in the United States: disparity among children of different ethnicity and socioeconomic status. Laryngoscope.

[127] Sultan-Khan LP (2010). African American Women's Online Evaluation of the Breast Cancer Awareness and Prevention Portal of the www.divahealth.org Website: Using Personal-Level Data and Website Ratings to Tailor and Improve the Portal [dissertation].

[128] Taylor KL, Davis JL, Turner RO, Johnson L, Schwartz MD, Kerner JF, Leak C (2006). Educating African American men about the prostate cancer screening dilemma: a randomized intervention. Cancer Epidemiol Biomarkers Prev.

[129] Thompson D, Baranowski J, Cullen K, Baranowski T (2007). Development of a theory-based internet program promoting maintenance of diet and physical activity change to 8-year-old African American girls. Comput Educ.

[130] Thornberry J, Bhaskar B, Krulewitch CJ, Wesley B, Hubbard ML, Das A, Foudin L, Adamson M (2002). Audio computerized self-report interview use in prenatal clinics: audio computer-assisted self interview with touch screen to detect alcohol consumption in pregnant women: application of a new technology to an old problem. Comput Inform Nurs.

[131] Thornberry JS, Murray KB, El-Khorazaty MN, Kiely M (2010). Acceptance, Communication Mode and Use of Audio Computer-Assisted Self Interview Using Touchscreen to Identify Risk Factors among Pregnant Minority Women. Methods Rep RTI Press.

[132] Tirado M (2011). Role of mobile health in the care of culturally and linguistically diverse US populations. Perspect Health Inf Manag.

[133] Tomita MR, Mann WC, Fraas LF, Burns LL (1997). Racial differences of frail elders in assistive technology. Assist Technol.

[134] Tran BQ, Buckley KM, Bertera EM, Gonzales PL (2009). Benefits & barriers to adoption of health IT in an elderly low-income, minority community-based environment. Conf Proc IEEE Eng Med Biol Soc.

[135] Trief PM, Teresi JA, Eimicke JP, Shea S, Weinstock RS (2009). Improvement in diabetes self-efficacy and glycaemic control using telemedicine in a sample of older, ethnically diverse individuals who have diabetes: the IDEATel project. Age Ageing.

[136] Valdez A, Banerjee K, Fernandez M, Ackerson L (2001). Impact of a multimedia breast cancer education intervention on use of mammography by low-income Latinas. J Cancer Educ.

[137] Vargas PA, Robles E, Harris J, Radford P (2010). Using information technology to reduce asthma disparities in underserved populations: a pilot study. J Asthma.

[138] Vincent D, Clark L, Zimmer LM, Sanchez J (2006). Using focus groups to develop a culturally competent diabetes self-management program for Mexican Americans. Diabetes Educ.

[139] Walker EA, Schechter CB, Caban A, Basch CE (2008). Telephone intervention to promote diabetic retinopathy screening among the urban poor. Am J Prev Med.

[140] Waters EA, Sullivan HW, Finney Rutten LJ (2009). Cancer prevention information-seeking among Hispanic and non-Hispanic users of the National Cancer Institute's Cancer Information Service: trends in telephone and LiveHelp use. J Health Commun.

[141] Weinstock RS, Izquierdo R, Goland R, Palmas W, Teresi JA, Eimicke JP, Shea S, IDEATel Consortium (2010). Lipid treatment in ethnically diverse underserved older adults with diabetes mellitus: statin use, goal attainment, and health disparities in the informatics for diabetes education and telemedicine project. J Am Geriatr Soc.

[142] Weinstock RS, Teresi JA, Goland R, Izquierdo R, Palmas W, Eimicke JP, Ebner S, Shea S, IDEATel Consortium (2011). Glycemic control and health disparities in older ethnically diverse underserved adults with diabetes: five-year results from the Informatics for Diabetes Education and Telemedicine (IDEATel) study. Diabetes Care.

[143] West SP, Lagua C, Trief PM, Izquierdo R, Weinstock RS (2010). Goal setting using telemedicine in rural underserved older adults with diabetes: experiences from the informatics for diabetes education and telemedicine project. Telemed J E Health.

[144] Wilkin HA, Valente TW, Murphy S, Cody MJ, Huang G, Beck V (2007). Does entertainment-education work with Latinos in the United States? Identification and the effects of a telenovela breast cancer storyline. J Health Commun.

[145] Wood RY, Duffy ME, Morris SJ, Carnes JE (2002). The effect of an educational intervention on promoting breast self-examination in older African American and Caucasian women. Oncol Nurs Forum.

[146] Wood FB, Sahali R, Press N, Burroughs C, Mala TA, Siegel ER, Rambo N, Fuller SS (2003). Tribal connections health information outreach: results, evaluation, and challenges. J Med Libr Assoc.

[147] Woolf SH, Johnson RE, Fryer GE, Rust G, Satcher D (2004). The health impact of resolving racial disparities: an analysis of US mortality data. Am J Public Health.

[148] Wright E, Fortune T, Juzang I, Bull S (2011). Text messaging for HIV prevention with young Black men: formative research and campaign development. AIDS Care.

[149] Yost KJ, Webster K, Baker DW, Choi SW, Bode RK, Hahn EA (2009). Bilingual health literacy assessment using the Talking Touchscreen/la Pantalla Parlanchina: Development and pilot testing. Patient Educ Couns.

[150] Shaw SJ (2010). The logic of identity and resemblance in culturally appropriate health care. Health (London).

[151] Crilly JF, Keefe RH, Volpe F (2011). Use of electronic technologies to promote community and personal health for individuals unconnected to health care systems. Am J Public Health.

[152] IQ Solutions, Inc (2001). US Department of Health and Human Services, Office of Minority Health.

[153] HHS Interagency Workgroup for the NHQR/NHDR (2011). Agency for Healthcare Research and Quality.

[154] Jimison H, Gorman P, Woods S, Nygren P, Walker M, Norris S, Hersh W (2008). Agency for Healthcare Research and Quality.

[155] Gruman JC (2011). Making health information technology sing for people with chronic conditions. Am J Prev Med.

[156] Rudd RE, Anderson E, Oppenheimer S, Nath C, Comings JP, Garner BP, Smith CA (2007). Health literacy: an update of medical and public health literature. Comings J, Garner B, Smith C, editors. Review of Adult Learning and Literacy, volume 7: Connecting Research, Policy, and Practice (National Center for the Study of Adult Learning and Literacy Series).

[157] (2011). Agency for Healthcare Research and Quality.

[158] Barrera M, Castro FG, Strycker LA, Toobert DJ (2012). Cultural adaptations of behavioral health interventions: a progress report. J Consult Clin Psychol.

[159] Brown M (2009). LGBT aging and rhetorical silence. Sex Res Social Policy.

[160] Daley A, Solomon S, Newman PA, Mishna F (2008). Traversing the margins: intersectionalities in the bullying of lesbian, gay, bisexual and transgender youth. J Gay Lesbian Soc Serv.

[161] Ivanitskaya L, O'Boyle I, Casey AM (2006). Health information literacy and competencies of information age students: results from the interactive online Research Readiness Self-Assessment (RRSA). J Med Internet Res.

[162] Chavez EL, Oetting ER (1995). A critical incident model for considering issues in cross-cultural research. Failures in cultural sensitivity. Int J Addict.

[163] Zanchetta MS, Poureslami IM (2006). Health literacy within the reality of immigrants' culture and language. Can J Public Health.

[164] DeCarlo P (2003). AIDS Partnership California.

[165] Chang VW, Lauderdale DS (2009). Fundamental cause theory, technological innovation, and health disparities: the case of cholesterol in the era of statins. J Health Soc Behav.

[166] Mangold WG, Faulds DJ (2009). Social media: the new hybrid element of the promotion mix. Bus Horiz.

[167] Pearson ML, Mattke S, Shaw R, Ridgely MS, Wiseman SH (2007). Agency for Healthcare Research and Quality.

