# Supplementary material for: Health and Wellness Technology Use by Historically Underserved Health Consumers: Systematic Review
Source: J Med Internet Res. 2012 May 31;14(3):e78. doi: 10.2196/jmir.2095 (PMC3799608; doi:10.2196/jmir.2095)
Supplement: Supplementary file 1 [file jmir_v14i3e78_app1.pdf]

Study Name	Author, Year (Reference)	Purpose (To determine...)	Key Finding
Health and nutrient content claims in food advertisements on Hispanic and mainstream prime-time television	Abbatangelo-Gray et al., 2008 [32]	To characterize frequency and type of nutrient and health claims on prime-time weeknight Spanish and English television advertisements	Spanish-language television aired more health and nutrition information than English-language television which may
A mass mediated intervention on Hispanic live kidney donation.	Alvaro et al., 2010 [33]	To determine the impact of a Spanish language mass media campaign on attitudes and behaviors referring to living organ donors.	The use of television and radio campaigns to improve Hispanics feelings and behaviors towards living organ donors.
Assessing the Cultural Relevance of an Education Program for Urban African Americans with Diabetes	Anderson et al., 2000 [34]	To determine the cultural relevance of an education program about diabetes for African Americans	The videotape vignettes were culturally relevant and effective.
Project ECHO: linking university specialists with rural and prison-based clinicians to improve care for people with chronic hepatitis C in New Mexico.	Arora et al., 2007 [35]	To determine the benefit of project ECHO which is a telemedicine and distance-learning program designated to improve access to quality health care for New Mexicans with Hepatitis C.	Project ECHO's teleconferencing, videoconferencing, internet-based assessment tools, online presentation, and telephone, fax, and email communications that connected specialists with primary-care providers in prisons and rural areas helped promote the use of best practices.
The Use of Quality Improvement and Health Information Technology Approaches to Improve Diabetes Outcomes in African American and Hispanic Patients	Baig et al., 2010 [36]	To review the effectiveness of health IT in health care interventions to improve diabetes process of care and outcomes in African American and Hispanics	No studies were found using health IT applications to enhance patient communication with providers, facilitate shared decision-making, or ones that emphasized culturally competent interactions with patients.
A pilot study of diabetes education via telemedicine in a rural underserved community--opportunities and challenges: a continuous quality improvement	Balamurugan et al., 2009 [37]	To look at the effects of a pilot study that implemented telemedicine technology for diabetes self-management education (DSME) for people in underserved rural communities.	The DMSE-T offers opportunities for DSME in rural areas.

process.			
Is There a Digital Divide among Physicians? A Geographic Analysis of Information Technology in Southern California Physician Offices	Bell et al., 2003 [38]	To determine whether physician offices in low income neighborhoods have different access levels to information technology than those offices in higher-income areas.	Primary care offices in poor and minority communities had high levels of access to and interest in web-based systems.
A study of the receptivity to telecare technology in a community-based elderly minority population	Bertera et al., 2007 [39]	To determine the willingness of elderly minority to use a variety of technology for telecare	Participants were most accepting of new technology when it would improve communication with a doctor or nurse
Promoting mealtime communication between adolescent mothers and their infants through videotape.	Black and Teti, 1997 [40]	To determine the effectiveness of a 15-minute culturally sensitive videotape about mealtimes for African-American mothers of infants.	The video was effective in creating mothers that are more involved with their infant.
Where's Maria? A video to increase awareness about breast cancer and mammography screening among low-literacy Latinas.	Borrayo, 2004 [41]	To discuss the need for a culturally competent intervention for Latina women surrounding Breast Cancer and the way in which an 8 minute Entertainment-Education soap opera formatted video was created for this intervention	Psychological barriers of Latina women that cause them to have low screening rates for breast cancer can be decreased by targeting certain attitudinal and behavioral issues in the community.
A PDA Intervention to Sustain Smoking Cessation in Clients With Socioeconomic Vulnerability	Buchanan and Khazanchi, 2010 [42]	To describe the acceptance, portability, technical difficulty, satisfaction and use time of PDAs as a tool to quit smoking	The PDA is useful to the task of quitting smoking but was not easy to use
A randomized control trial evaluating the educational effectiveness of a rapid HIV posttest counseling video.	Calderon et al., 2009 [43]	To determine if a 15-minute educational video (in either Spanish or English) is as effective as a 20 minute session with an HIV counselor	The video was found to be at least as effective as the session with an HIV counselor.

La Clínica del Pueblo: A Model of Collaboration Between a Private Media Broadcasting Corporation and an Academic Medical Center for Health Education for North Carolina Latinos	Calles-Escandon et al., 2009 [44]	To determine the ability of *La Clinica del Pueblo* to disseminate culturally appropriate health information through use of weekly radio and corresponding newspaper column	Listenership to La Clinica del Pueblo had a large increase over the 200 consecutive weeks of playing; statistical increase in medical knowledge
A tailored multimedia nutrition education pilot program for low-income women receiving food assistance.	Campbell et al., 1999 [45]	To describe the development and findings from a pilot test of a multimedia tool used to improve dietary behaviors in low income women in the Food Stamp program	The computer based multi-media tool's use of soap opera's and interactive infomercials was effective in improving knowledge, state of change, and certain eating behaviors.
A Patient-Centric, Provider-Assisted Diabetes Telehealth Self-management Intervention for Urban Minorities	Carter et al, 2011 [46]	To describe the implementation of an online self-management intervention program for African Americans with diabetes	An online diabetes self-management culturally competent program coupled with a biweekly meeting with a virtual nurse can help improve health outcomes for African Americans
Comparison of three interventions to increase mammography screening in low income African American women.	Champion et al, 2006 [47]	To compare three types of intervention (pamphlet, culturally appropriate video, and interactive computer-assisted instruction program) for African Americans on the importance of breast cancer screening. Note: storytelling by a famous African American person was used in both the video and computer-assisted instruction program	The tailored approach of the interactive computer-assisted instruction program was most effective showing that a tailored program is better than targeted messages (in print or video format). Further, interactive interventions are more effective than non-interactive interventions
Interpreter Services in Emergency Medicine	Chan et. al., 2010 [48]	To explore the pros and cons of different ways to communicate with non-English speakers in the emergency department of a hospital	Professional interpreters, ad hoc interpreters, telephone interpretation services, and bilingual/multilingual staff can be used to interpret for patients who do not speak English in the emergency room.
Relationship between health service use and health information technology use among older adults: analysis of the US National Health Interview Survey.	Choi, 2011 [49]	To examine if older adults use of health IT and health related internet activities differed from their younger counterparts	Older adults that use general health services were more likely to use health IT and the internet for health related activities than those who did not use general health services. Further, the access gap was worse for racial/ethnic minority older

			adults, poorly educated older adults, and older adults with lower socio-economic status.
Provision of pharmacy services to underserved populations via remote dispensing and two-way videoconferencing.	Clifton et al., 2003 [50]	To discuss the effectiveness of a telepharmacy program that dispenses low -cost medications through use of remote dispensing and counseling.	The program was effective and a majority of the patients were satisfied with the time required to obtain the medications and counseling via the videoconferencing
Telehealth for elders and their caregivers in rural communities.	Coen et al., 2002 [4]	To discuss the pros and cons of using telehealth for rural elderly.	Telehealth can create a more comfortable way for elders to communicate with health professionals along with help them not feel as isolated
Health Information Technology: Addressing Health Disparity by Improving Quality, Increasing Access, and Developing Workforce	Custodio et al, 2009 [17]	To examine the positive effect EHRs can have not only on minority groups but on the population as a whole	EHRs have a favorable impact on quality of care, patient safety, and system delivery. Further, they can help better aid minority populations.
The impact of a culturally appropriate STD/AIDS education intervention on black male adolescents' sexual and condom use behavior.	DeLamater et al., 2000 [51]	To determine if an educational video for 15-19 year old black males about HIV risk and sexual behaviors would improve health behaviors	The educational video was not effective.
Evaluation, use, and usefulness of prescription drug information sources among Anglo and Hispanic Americans.	Delorme et al., 2010 [52]	To determine the differences in how Hispanics and Anglos use and evaluate advertising and mediated sources of prescription drug information.	Hispanics use doctors, internet sources, and direct to consumer advertising (DTCA) for sources of information while Anglos use health websites and health care professionals. Further, Anglos are more likely to use health websites like WebMD while Hispanics rely on television and DCT TV advertisements and like having Spanish language media and healthcare

			professionals.
The effect of prevention messages and self-efficacy skill building with inner-city women at risk for HIV infection.	DeMarco et al., 2009 [53]	To examine if gender-sensitive and culturally relevant HIV prevention films combined with self-efficacy and skill building exercises can positively influence safe sex for inner city women.	The intervention led to self-reported safer sex behaviors.
Culturally relevant HIV interventions: transcending ethnicity.	DeMarco and Norris, 2004 [54]	To discuss the pretesting of a culturally relevant film for African American and Latina teen girls about HIV.	The people that were pretested found the messages of the video to be strong, especially those who shared gender identity and socioeconomic status.
The Digital Divide at an Urban Community Health Center: Implications for Quality Improvement and Health Care Access	Denizard-Thompson et al., 2011 [55]	To determine the number of clinic patients that use the internet and cell phones and to determine their interest in using these technologies for health purposes (managing clinical appointments and patient education)	African Americans were more likely than whites and females more likely than males to use text messages. Also the patient's interest in using the technologies was high.
Developing the Native People for Cancer Control Telehealth Network.	Doorenbos, 2011 [56]	To discuss the challenges and opportunities of implementing a telehealth network for delivering post diagnosis cancer care clinical services and education to American Indian and Alaska Native patients, their families, and healthcare providers.	Overall the telehealth network was a success. Some of the keys to success that were identified were resolution of technical issues, provider and community acceptance, working closely with members of the community, understanding tribal government, and addressing cultural competency issues. Lack of communication and collaboration were often causes of IT implementation failure.
Disparities by Race and Ethnicity in Cancer Survivor Stories Available	Eddens et al., 2009 [57]	To examine the availability of cancer survivor studies of ethnic minorities online.	Racial minorities were underrepresented, especially those of a Hispanic descent.

on the Web.			
Strategies to prevent HIV transmission among heterosexual African-American men.	Essien et al., 2005 [58]	To discuss the findings of focus groups run in order to develop a culturally sensitive videotape-based HIV prevention for heterosexual African American men.	The focus groups found that the biggest risk factor is that African American men have high sexual risk behaviors (not using a condom) and substance abuse issues. Interventions should address condom use, condom availability, correct condom use, eroticizing condoms, and substance abuse preventions. Further, the videotaped messages should include pieces of sensational effects of the disease.
Mobile Phone Technology: A New Paradigm for the Prevention, Treatment, and Research of the Non-sheltered "Street" Homeless?	Eyrich-Garg, 2010 [59]	To examine the number of homeless people who do not use the shelter system but own mobile phones and to examine how they to effectively use their mobile phones	Half (44%) of the sample owned mobile phone. Of those who owned a phone 100% of them made or received a call in the last 30 days, 61% of them sent or received a text, and 20% of them accessed the internet.
Comparison of assisted reproductive technology utilization and outcomes between Caucasian and African American patients in an equal-access-to-care setting	Feinberg et al., 2006 [60]	To determine the reasons for the disparity existing for minorities using assisted reproductive technology (ART)	African American women utilized ART services more when access to care was improved.
Exploring feasibility of home telemanagement in African Americans with congestive heart failure.	Finkelstein et al., 2010 [61]	To determine the feasibility of using a Home Automated Telemanagement for African Americans with congestive heart failure who don't have prior computer experience.	There was a high level of acceptance of the Home Automated Telemanagement system.
Health information technology and quality improvement for community health centers	Fiscella and Geiger, 2006 [13]	To show why HITs should be federally supported at community health centers in order to improve quality control.	Four reasons for slow HIT diffusion are cost, complex selection process, misaligned costs and benefits, and time-consuming implementation. The benefits of HIT adoption for the HHS are reduction

			in costs, better patient data, and equity of HIT diffusion.
How Innovative Treatment Models and Data Use are Improving Diabetes Care among Older African American Adults	Fitzner et al., 2011 [62]	To address strategies (such as telehealth to improve health outcomes of older African Americans with diabetes.	Telehealth (over traditional methods) created more frequent communication with patients, increased ability to serve the underserved, more timely and accurate monitoring, and improved management of data. However, its downfall was technological problems and a lack of physical contact with patients.
Using decision aids in community-based primary care: a theory-driven evaluation with ethnically diverse patients	Frosch et al., 2008 [63]	To access the benefits of informational brochures and video decision aids for cancer patients in a racially diverse sample	Patients that used the video decision aid had a larger knowledge base and were more likely to want to be the primary decision-maker.
The development of SisterTalk: a cable TV-delivered weight control program for black women.	Gans et al., 2003 [64]	To discuss the development of SisterTalk, a cable television intervention for African American's dealing with obesity.	A list of issues addressed while trying to culturally tailor a video are listed.
Quality in medical translations: a review.	Garcia-Castillo and Getters, 2007 [65]	To conduct a systematic literature review of the World Wide Web and electronic library resources on clinical and medical research document translations.	Health translations are more complicated than mechanically converting one language to another. Cultural equivalency is a real issue that needs to be examined when translating health literature.
Pre-experience perceptions about telemedicine among African Americans and Latinos in South Central Los Angeles	George et al., 2009 [12]	To explore the feelings towards telemedicine of the African American and Latino populations in South and Central California	The concerns about telemedicine varied between the African Americans and Latinos. Therefore, the two groups must be accessed separately.
Use of health information technology among racial and ethnic underserved communities	Gibbons, 2011 [9]	To examine health IT use among racial and ethnic minorities	Discusses barriers and challenges to using health IT to better the health related quality of life for racial and ethnic minorities

	Theorizing the digital divide: Information and communication technology use frameworks among poor women using a telemedicine system	Gilbert et al., 2008 [66]	To examine how best to approach the digital divide in order to implement information and communication technology (ICT)	In order to approach this problem, policy needs to change and literacy needs to be examined.
	Health-related characteristics and preferred methods of receiving health education according to dominant language among Latinos Aged 25 to 64 in a large Northern California health plan	Gordon and Iribarren, 2008 [67]	To examine how Spanish dominant (SD), English dominant (ED) and bilingual (BIL) Latinos differ from their counterparts in their preference and access to Health IT	SD and BIL Latinos had lower internet and computer access than their ED counterparts. Latinos with lower English proficiency wanted health education through non-internet modalities such as videos, telephone messages, Spanish print materials, and small health groups.
	The effect of breast cancer screening messages on knowledge, attitudes, perceived risk, and mammography screening of African American women in the rural South.	Grindel et al., 2004 [68]	To determine the effect of three different types of breast cancer screening messages on knowledge, attitudes, perceived risk of the disease, and mammography screening: positive/upbeat, neutral/cognitive, and negative/fear	Health experts and nurses should aim to design interventions that create positive attitudes, increase knowledge about breast cancer screening, and encourage women to get screened; interventions need to be done in context to cultural norms
	Racial differences in attitudes toward innovative medical technology	Groeneveld et al., 2006 [69]	To examine the racial differences in innovativeness and innovativeness with medical technology	African Americans and Whites have different attitudes towards medical innovation with Whites being more open to trying new medical technologies
	Counseling and Motivational Videotapes Increase Duration of Breast-Feeding in African-American WIC Participants Who Initiate Breast-Feeding	Gross et al., 1998 [70]	To evaluate the effect of using motivational videotapes or peer counseling on African American women who are breast feeding	Peer counseling and motivational videos had a positive effect on the women and encouraging them to breast feed.
	Learning about assistive technology: Hispanics and a national sample.	Guzman, 2008 [71]	To determine if there are differences (both inter group and intra group) among Hispanics and how they learn about assistive technology	Regardless of ethnic/racial group, assistive technologies are often taught by doctors and medical professionals; some differences were found in the use of extended family, friends, neighbors, and the internet as information sites

Hispanics' Awareness of Assistive Technology	Guzman and Ostrander, 2009 [72]	To compare Hispanics' awareness of programs that help them acquire Assistive Technology and whether they actually seek out the Assistive Technology	A large disparity exists between the awareness of Assistive Technology and the receiving of Assistive Technology
Use of the internet to obtain cancer information among cancer patients at an urban county hospital.	Helft et al., 2005 [73]	To evaluate rates, predictors, and barriers to obtaining cancer information on the internet.	While many cancer patients wished to use the internet to research cancer, the interventions online are not accessible, trustworthy, and understandable for the given population. Further, less education, holding an African American identity, and female sex were associated with lower understanding of internet information.
Influence of communicator's race on efficacy of an HIV/STD prevention intervention among African American and Caucasian college students.	Helion et al., 2008 [74]	To examine the influence of the communicator's race on HIV and STD educational videos efficacy in African-American and Caucasian college females	No effect of race of communicator for either African-Americans or Caucasians
Food advertising in the age of obesity: content analysis of food advertising on general market and African American television.	Henderson and Kelly, 2005 [75]	To document nutritional claims and types of food advertized on television programming directed towards African-Americans	In addition to more food advertisements, there were more advertisements for fast food, candy, soda, or meat; more claims were related to fat content in comparison to the lean claims maid in general market advertisements
Computer expert systems for African-American smokers in physicians offices: A feasibility study	Hoffman et al., 2006 [76]	To access acceptability of a computer interactive feedback system coupled with stress reduction audiotapes to help reduce disparities in morbidity and mortality from smoking-related diseases in African Americans	The stage-tailored computer interactive feedback plus stress reduction intervention was successful.
A multidimensional view of personal health systems for underserved populations.	Horan et al., 2010 [77]	To assess the organizational requirements needed to implement PHRs in undeserved communities.	Health information technology and political reforms are needed to increase access to PHRs.
Culturally appropriate storytelling to improve blood pressure: a	Houston et al., 2011 [78]	To evaluate an interactive storytelling intervention that is played through use of a DVD	Storytelling method worked well in patients with baseline uncontrolled hypertension

randomized trial.			
"Let's Talk About Sex": pilot study of an interactive CD-ROM to prevent HIV/STIS in female adolescents.	Ito et al., 2008	To develop and test an interactive CD-ROM on the prevention of STIs	Hispanic and African-American adolescents chose a host of their same race/ethnicity on the CD-ROM; the CD-ROM was successful in increasing knowledge
Using computer-based assessments to evaluate interactive multimedia nutrition education among low-income predominantly Hispanic participants.	Jantz et al., 2002 [79]	To determine the effectiveness of interactive multimedia (IMM) for low-income Hispanic persons	Computer-based evaluations can be especially valuable for low-income and low-literate populations.
Electronic health records: Use, barriers and satisfaction among physicians who care for black and Hispanic patients	Jha et al., 2009 [80]	To determine whether providers serving minorities adopt EHR at a similar rates to their counterpart providers	There was no significant evidence that providers serving minorities had lower EHR adoption rates, faced different barriers to EHR adoption or were less satisfied with EHRs.
Effectiveness of a video-based motivational skills-building HIV risk-reduction intervention for inner-city African American men	Kalichman et al., 1999 [81]	To report the effectiveness of a 6 hr video based motivational skills intervention and a 6 hr video based contact matched HIV education	The motivational skills intervention worked better at increasing condom use
Closing the digital divide in HIV/AIDS care: development of a theory-based intervention to increase Internet access	Kalichman et al., 2002 [82]	To describe the development of an AIDS intervention for people with no prior experience with the internet.	The pilot testing found the workshop-style intervention as a viable method to help mitigate the digital divide in HIV/AIDS care
Disparities in usage of assistive technology among people with disabilities.	Kaye et al., 2008 [83]	To examine the differences in technology usage of subpopulations of people with disparities.	People with lower access and use of assistive devices were found to be those with lower education, racial/ethnic minorities, lower income, and later onset of disability or having a mental disability.
Effects of a videotape to increase use of poison control	Kelly et al., 2003 [84]	To examine the impact of informative videotapes on the knowledge, attitudes, behaviors, and behavioral	Videotape intervention was highly effective in positively influencing knowledge, attitudes,

centers by low-income and Spanish-speaking families: a randomized, controlled trial.		intention regarding the use of poison control centers (PCC) in low-income minority and speaking-speaking parents	behaviors, and behavioral intentions
Racial/Ethnic Disparities in the Utilization of High-Technology Hospitals	Kim et al., 2010 [85]	To determine if ethnic and racial minority patients are less likely to use high technology hospitals.	Hispanic patients were less likely to use high-technology hospitals than their white counterparts when other factors were controlled.
Comparing narrative and informational videos to increase mammography in low-income African American women	Kreuter et al., 2010 [86]	To compare outcomes of using narrative verse informational videos for cancer-related beliefs.	Narrative video was not only better received than the informational video but also increased the effectiveness of intervention to reduce cancer health disparities
LUCHAR: Using Computer Technology to Battle Heart Disease Among Latinos	Leeman-Castillo et al., 2010 [87]	To evaluate a new design of a community-based health kiosk for both English and Spanish speaking Latinos.	Significant improvements in nutrition and physical activity were observed while no changes were found in smoking behaviors.
Acceptance and expectations of information technology to support hypertension self-care in African Americans: a qualitative inquiry.	Li et al., 2007 [88]	To explore African American patients' perspective on benefits of technology use	Technological interventions are more likely to succeed if comprehensive multidisciplinary support in a personalized, user friendly way are provided.
Patient Panel of Underserved Populations and Adoption of Electronic Medical Record Systems by Office-Based Physicians	Li and West-Strum, 2010 [16]	To examine the connection between the adoption of EMRs and patient panels of underserved populations seen by physicians	Physicians who see high levels of Hispanic patients have lower access to EMRs with essential functionalities.
Racial disparities in health information access: resilience of the digital divide	Lorence et al., 2006 [89]	To examine if policy initiatives set in place in the 1990s eliminated the information "digital divide".	Between 2000 and 2002, the divide between computer usage Hispanics and Whites and between African-Americans and Whites lessened while the gaps in internet usage stayed the same. Further, there was a disparity in health information seeking between these populations.

Social equity and access to the world wide web and e-mail: Implications for design and implementation of medical applications	Mandl et al., 1998 [90]	To measure the predictors, rates, and types of access to web based medical services.	Income directly correlates to internet access and internet access is unevenly distributed across races.
Designing e-health interventions for low-health-literate culturally diverse parents: addressing the obesity epidemic.	Mackert et al., 2009 [91]	To determine the ability of e-health to educate low-health-literate audiences on the topic of obesity	E-health can be used as an effective education tool for low-health-literate audiences but the interventions must be very usable in order to meet the different levels of health literacy.
Will decreasing assisted reproduction technology costs improve utilization and outcomes among minority women	McCarthy-Keith et al., 2010 [92]	To evaluate assisted reproduction technology (ART) use and outcomes of minority women.	African American women had a fourfold increase in ART usage while Hispanic women had a decrease. However, pregnancy rates of African American women verse White women were still low. This shows that access is not enough to bridge the disparity.
Use of videotaped interactions during pediatric well-child care: impact at 33 months on parenting and on child development.	Mendelsohn et al., 2007 [93]	To assess the impact of the Video Interaction Project (VIP) to promote early child development. VIP is a program where child development specialists use videotaped interactions to help promote development	VIP was associated with lower parental stress and VIP children were more less likely to have developmental delays and more likely to have normal cognitive development
Health Information Websites: characteristics of US users by race and ethnicity	Miller et al., 2007 [94]	To determine how different race and ethnicities use health Websites	Non-Hispanic whites reported higher use of health Websites than African Americans or Hispanics. A stronger association between perceived health and Website usage was found for Whites and Hispanics while a stronger association between health literacy and usage was found for Hispanics. The relationship between education and usage was much stronger for African Americans and Hispanics than other groups.
Health Information Technology and Quality of Health Care: Strategies for Reducing Disparities in	Millery and Kukafka, 2010 [15]	To conduct a systematic literature review to identify outcomes of health IT in Underresourced settings (URSs).	There must be an organizational culture and IT champions in order for the health IT to be accepted

Underresourced Settings			
Use of MP3 players to increase asthma knowledge in inner-city African-American adolescents.	Mosnaim et al., 2008 [95]	Evaluate Adolescents' Disease Empowerment and Persistency Technology (ADEPT) on asthma knowledge through providing asthma information messages between music tracks on an MP3 player; messages were recorded by famous athletes, musicians, and other celebrities	The innovative way of disseminating Asthma knowledge information was effective
A content analysis of health and physical activity messages marketed to African American children during after-school television programming	Outley and Taddese, 2006 [96]	Examine the number of food advertisements African-American children see on children's television programs aired on primarily African-American stations	Of the numerous food and beverage commercials, only 8% had health-related content and only 9% had physical activity-related content
Impact of commercials on food preferences of low-income, minority preschoolers.	Nicklas et al., 2011 [97]	To determine the effectiveness of commercials about fruits and vegetables can have an impact on children's preferences for certain fruits and vegetables	The commercials may be an effective strategy for influencing African American and Hispanic children to eat fruits and vegetables.
Development and evaluation of a sexual decision-making and social skills program: "the choice is yours--preventing HIV/STDs".	Noell et al., 1997 [98]	To determine the effectiveness of an interactive video designed to reduce HIV/STD risk behavior.	Overall, the videos were effective. The interest level in the videos was due to the culturally tailored material. While the scenarios were similar for each group, the language, clothing, and interpersonal behaviors were different among the different races/ethnicities.
Promoting HIV risk awareness and testing in Latinos living on the U.S.-Mexico border: the Tú No Me Conoces social marketing campaign.	Olshefsky et al., 2007 [99]	To test the ability of the 'Tu No Me Conoces' social marketing campaign (Spanish-language radio, print media, web site, toll-free HIV testing referral hotline) on improving HIV awareness of Latinos living on the US-Mexican border.	The campaign resulted in increased HIV testing.

Marketing nutrition among urban Latinos: the SALUD! campaign.	Perez-Escamilla et al., 2000 [100]	To determine the ability of Salud!, a radio and print media campaign that targets Latino children and their caretakers , to improve understanding of nutrition.	The campaign was successful. Further, multiple media channels should be used as one single channel did not strongly dominate the coverage of the campaign.
An intervention to decrease cancer fatalism among rural elders.	Powe and Weinrich, 1999 [101]	To determine the effectiveness of a video intervention for rural elders (mostly African American) on the topic of colorectal cancer.	"Telling the Story...To Live in God's Will" was found to be an effect, cost-effective intervention to increase knowledge of colorectal caner and to decrease fatalism.
Impact of A Smoking Prevention Interactive Experience (ASPIRE), an interactive, multimedia smoking prevention and cessation curriculum for culturally diverse high-school students.	Prokhorov et al., 2008 [102]	To access the long-term efficacy of multimedia computer based smoking intervention for culturally diverse high-schoolers.	While the video lowered the smoking initiation rates among the participants, Hispanic teens were still likely to initiate smoking. This leads the authors to believe that attention should be put on the strong kinship bonds of the teens (familialismo, personalismo).
Finding Teens in Their Space: Using Social Networking Sites to Connect Youth to Sexual Health Services	Ralph et al., 2011 [103]	To examine the pros and cons of disseminating sex-health information to teens via social network sites (SNS).	Social Network Sites (SNS) have both positives and negatives when it comes to disseminating sexual health information. However, it has potential to reach diverse youth and should be looked at further as an outreach strategy.
Randomized controlled trial of telephone case management in Hispanics of Mexican origin with heart failure.	Riegel et al., 2006 [104]	To test the effectiveness of telephone case management for Hispanics of Mexican origin with heart failure. The study looked at decreasing hospitalizations, improving Health Related Quality of Life and decreasing depression.	While this type of telemedicine has worked for the general population, it was found to be ineffective for Hispanics that were so ill.
Digital divide: variation in internet and cellular phone use among women attending an urban sexually transmitted infections clinic.	Samal et al., 2010 [105]	To describe (1) internet, cell phone, and text message use, (2) the acceptability of health advice via these modalities, and (3) demographic characteristics associated with ICT use of women	All three modalities (internet, cell phone, text messages) were acceptable for health communication. Also, higher education and younger age were associated with usage in all three modalities.

		at an urban STI clinic	
Educational video tool to increase syphilis knowledge among black and Hispanic male patients	Sanchez et al., 2010 [106]	To determine the effectiveness of a certain educational video to increase knowledge on syphilis in at-risk Black and Hispanic males	The video was effective across all socio-demographic and across all men regardless of their sexual behaviors
Video Tool to Promote Knowledge of Syphilis Among Black and Hispanic Men Recruited From Clinical and Non-Clinical Settings	Sanchez et. al., 2010 [107]	To assess whether viewing the 5 minute "Syphilis and Men" video improved syphilis knowledge in men when increased risk of syphilis (minority men who are MSM)	The video helped increase syphilis knowledge amongst these men.
Evaluating a Bilingual Video to Improve Infant Feeding Knowledge and Behavior Among Immigrant Latina Mothers	Scheinmann et al., 2010 [108]	To determine if a bilingual English/Spanish infant feeding video would help decrease child obesity in Latino children of immigrant mothers.	Bilingual videos can have a positive impact on maternal knowledge and behaviors.
Seeing in 3-D: Examining the reach of diabetes self-management support strategies in a public health care system	Schillinger et al., 2008 [109]	To determine if a tailored self-management system (SMS) that works in multiple languages (English, Spanish, and Cantonese) paired with weekly automated telephone disease management (ATDM) or monthly group visits would reach patients in a safety net system.	ATDM resulted in higher engagement with those with limited English language proficiency and literacy having the highest engagement.
Caring for Hispanic patients interactively: simulations and practices for allied health professionals	Schitai, 2004 [110]	To determine the effectiveness of a new tool for training professionals about Hispanic Patients in a culturally competent way	The computer-based training (CHISPA) that includes a CD and website that focus on patient simulations, Spanish language and internet practices, and an activities module was effective in increasing cultural understanding, integrating real-world experiences with theory, and providing useful information.
Perceptions of factors influencing use of an electronic	Schnall et al., 2011 [111]	To determine the current perceptions of Case Managers (CMs) who	The CMs were worried about predisposing (system functionality and

record for case management of persons living with HIV		facilitate continuity of care for people living with HIV on using HIT	confidentiality), training, efficiency and quality of the systems. While HITs may still be used for continuity of care, these fears must be addressed
Disparity in assisted reproductive technologies outcomes in black women compared with white women	Seifer et al., 2008 [112]	To compare success rates of African-American and Caucasian women undergoing In Vitro Fertilization	African-Americans are less likely to seek IVF; Black race is an independent risk factor that is not explained by the characteristics looked at in the study
Trends of racial disparities in assisted reproductive technology outcomes in black women compared with white women: Society for Assisted Reproductive Technology 1999 and 2000 vs. 2004-2006.	Seifer et al., 2010 [113]	To continue the previous study (2008) but this time examine In Vitro Fertilization for years 2004-2006	While numbers improved slightly from 2000, African-Americans are still less likely to seek IVF and more likely to have fewer live births.
Information technology as a tool to improve the quality of American Indian health care.	Sequist et al., 2005 [114]	To describe the disparities for American Indians and the work being done by the Indian Health Service (IHS) to improve these disparities. Further, the article discusses how the IHS is using information systems to increase standardization and improve American Indian health.	The Indian Health Service (IHS) consists of three units: (1) federally operated direct care system (2) tribally operated care (3) urban care services. However, health disparities do exist. EHRs are being installed and should help address quality improvements
Implementation and use of an electronic health record within the Indian Health Service.	Sequist et al., 2007 [115]	To evaluate the implementation of Electronic Health Records in the Indian Health Service (IHS)	Overall clinicians were found to support the EHR technology but felt that the limited use of key functions of the technology led them to not be able to use the technology to its full potential.
Media influence on awareness and utilization of assisted reproduction technology in Hispanic populations.	Serou and Quintero, 2011 [116]	The letter to the editor was written to discuss a recent article about the utilization and outcomes of assisted reproduction technology in minority women (especially Hispanic women).	The authors noted that when cost is set aside, Hispanics are less likely to seek infertility treatments because of issues like language/cultural barriers, lack of provider communication, scheduling problems, and decreased availability of services.

The Informatics for Diabetes and Education Telemedicine (IDEATel) project.	Shea, 2007 [117]	To determine the effectiveness of telemedicine (specifically IDEATel) for mostly Hispanic and African Americans in New York city and upstate New York	Telemedicine helped improve glycemic control, blood pressure levels, and cholesterol levels and was found to be effective for managing diabetes in undeserved populations
Columbia University's Informatics for Diabetes Education and Telemedicine (IDEATel) Project: rationale and design.	Shea et al., 2002 [118]	To describe the IDEATel project's rationale and design.	The project consists of a video camera for voice conferencing with nurse managers, home glucose meter and blood pressure cuff that can take readings and upload the results to a computer database, an access point for patients to view their clinical data through graphical displays, secure messaging including email and a webpage that is in both English and Spanish.
Adoption of health information technology in community health centers: Results of a national survey	Shields et al., 2007 [119]	To determine the number of community health centers (CHCs) with health IT and EHRs	Nine-tenths of the CHCs stated lack of capital to invest in EHRs as a barrier to adoption, four-fifths stated inability to integrate EHR with the current system, and three-fourths stated concerns about the loss of productivity or income during transactions
Older Patients' Enthusiasm to Use Electronic Mail to Communicate With Their Physicians: Cross-Sectional Survey	Singh et al., 2009 [120]	To determine the enthusiasm levels of older adults in using email as a communication method with their physicians	African Americans and Hispanics were more enthusiastic than their Caucasian counterparts about using email to communicate with their physician.
Client satisfaction with rapid HIV testing: Comparison between an urban sexually transmitted disease clinic and a community-based testing center.	Smith et al., 2006 [121]	To determine if rapid HIV testing which allows same-day results is a positive improvement in HIV testing or if it makes people uncomfortable.	Latinos were more likely to state that the results were returned too quickly and that they would rather wait a week. Overall, the clients found the rapid results to be more stressful.
Asthma 1-2-3: a low literacy multimedia tool to educate African American adults about asthma	Sobel et al., 2009 [122]	To determine the effectiveness of using a video script combined with storyboard imagery to educate African-American adults on asthma	The tool developed is useful for future education

Latina breast cancer survivors informational needs: information partners	Sorensen et al., 2009 [123]	To access and understand how Latinas with breast cancer use informatics tools to manage their health.	Latinas find having a select support network of people a necessity and therefore e-health and informatics should focus on networks rather than individuals.
Racial disparity and technology diffusion: The case of cardioverter defibrillator implants, 1996-2001	Stanley et al., 2007 [124]	To determine the racial disparities of elderly people using implantable cardioverter defibrillators.	Elderly black patients were less likely to use implantable cardioverter defibrillators (ICDs) than their white counterparts. However, the disparity declined by 40% over the 3 years of the study.
A program of interventions designed to increase mammography rates in women ages 50 years and older for an underserved racial minority	Stelger et al., 2003 [125]	To determine if the intervention strategies used for African-American women who were being encouraged to get a mammography in Philadelphia worked	The intervention that consisted of a video, educational mailings, posters, prepaid postcards, and public service messages most likely worked. However, due to limited responses, the researchers cannot make hard conclusions.
Recent epidemiology of pediatric cochlear implantation in the United States: Disparity among children of different ethnicity and socioeconomic status	Stern et al., 2005 [126]	To determine if SEC and race/ethnicity play a part in the number of children provided with cochlear implantation.	White and Asian children were more likely to be implanted than Hispanic and African-American children.
African American women's online evaluation of the breast cancer awareness and prevention portal of the www.divahealth.org website: using personal-level data and website ratings to tailor and improve the portal	Sultan-Khan, 2010 [127]	To conduct a survey to evaluate a new Breast Cancer Awareness and Prevention Portal for African American Women as an additional resource to the www.divhealth.org website	Income and valuing the website features were found to be significantly correlated with website rating. Overall, the website is a viable method to provide preventative health information to this population.
Educating African American men about the prostate cancer screening dilemma: a randomized intervention.	Taylor et al., 2006 [128]	To determine the effect of educational interventions on knowledge, decisional conflict over whether or not to get screened, satisfaction with decision to do a screening, and self-reported screening; intervention was provided through a video or a	Being given either the video or booklet (as opposed to nothing like the control group) increased screening rate and knowledge and decreased the decisional conflict over whether or not to get screened, but no difference was found between the two

		printed booklet	types of media
Development of a theory-based internet program promoting maintenance of diet and physical activity change to 8-year-old African American girls.	Thompson et al., 2007 [129]	To determine the effectiveness of a theory-based internet and multimedia program that promotes healthy eating and physical activity for 8 year old African American girls	The multimedia tool that utilizes the internet and integrates theories is a viable method for changing diets and physical activity of 8 year old African American girls.
Audio computerized self-report interview use in prenatal clinics	Thornberry et al., 2002 [130]	To determine if computer interviewing women about their alcohol consumption at a prenatal clinic is a viable way to reach undeserved women	Even though many of them women were disadvantaged (24% did not complete high school, 43% unemployed, 30% received public assistance, 59% used computers occasionally or never), 96% of the women stated that the computer was not difficult to use and 90% liked answering the questions via the computer
Acceptance, Communication Mode and Use of Audio Computer-Assisted Self Interview Using Touchscreen to Identify Risk Factors among Pregnant Minority Women	Thornberry et al., 2010 [131]	To determine the acceptability, communication mode, and use of Audio Computer Assisted Self Interview (A-CASI) to change risk behaviors in pregnant women.	A-CASI was accepted amongst the women with African American's enjoying the experience the most.
Role of mobile health in the care of culturally and linguistically diverse US populations.	Tirado, 2011 [132]	To review public policy on four areas: mobile health service access and duty of care of physicians, affordability of mobile health, protocols for health data collection and distribution on mobile phones, and cultural and linguistic appropriateness of mobile health	Policy recommendations are listed. However, the question posed is whether policy makers should plan ahead for these challenges or wait until the technology exist before taking action.
Racial differences of frail elders in assistive technology.	Tomita et al., 1997 [133]	To compare the differences in Black and White elderly patients living at home.	African Americans had fewer home modifications (most likely because they often rent their residencies)

		Specifically the study looked at assistive devices and home modifications.	and had fewer assistive devices that their white counterparts.
Benefits & barriers to adoption of health IT in an elderly low-income, minority community-based environment	Tran et al., 2009 [134]	To discuss the attitudes, benefits, and barriers to implementation of health IT	Despite challenges to the adoption of technology, senior residents will adopt the technology if they understand the benefits of the technology which includes keeping their independence
Improvement in diabetes self-efficacy and glycaemic control using telemedicine in a sample of older, ethnically diverse individuals who have diabetes: the IDEATel project.	Trief et al., 2009 [135]	To determine whether self efficacy in older diabetic patients can relate to better health outcomes such as gycaemic control.	Diabetes self efficacy was linked to improving glycaemic control.
Impact of a multimedia breast cancer education intervention on use of mammography by low-income Latinas.	Valdez et al., 2001 [136]	To determine the effectiveness of a multimedia (video, animation, still photos, music, and sound on a kiosk) made up of ten modules on educating women on breast cancer	The kiosks were effective as 40% of the women who had or scheduled a mammography after the intervention stated that the intervention was the reason they made the appointment
Using Information Technology to Reduce Asthma Disparities in Underserved Populations: A Pilot Study	Vargas et al., 2010 [137]	To design and evaluate an interactive computer questionnaire regarding asthma symptoms of children whose parents have either low health literacy and/or limited English proficiency.	The reliability of the computer based questionnaire was higher than that of a pen and pencil questionnaire.
Using focus groups to develop a culturally competent diabetes self-management program for Mexican Americans.	Vincent et al., 2006 [138]	To describe factors that help or hinder diabetes self-management and to use these factors to create a culturally component diabetes self-management program.	The culturally competent diabetes self management program should contain a family aspect and include techniques for self management and diet modification. Further, information should be delivered in a few different methods.
Telephone intervention to promote diabetic retinopathy screening among the urban poor	Walker et al., 2008 [139]	To determine if a bilingual tailored telephone intervention for an urban minority diabetes population would result in higher screening rates for	The intervention was successful, especially among subjects who had poor diabetes control.

		retinopathy than a print intervention.	
Cancer Prevention Information-Seeking Among Hispanic and Non-Hispanic Users of the National Cancer Institute's Cancer Information Service: Trends in Telephone and LiveHelp Use	Waters et al., 2009 [140]	To examine cancer prevention via a telephone service and an instant messaging service (LiveHelp)	LiveHelp was utilized more by non-Hispanics than Hispanics. Half of the Hispanics who utilized the cancer prevention techniques did so in Spanish (although LiveHelp was not available in Spanish).
Lipid treatment in ethnically diverse underserved older adults with diabetes mellitus: statin use, goal attainment, and health disparities in the informatics for diabetes education and telemedicine project.	Weinstock et al., 2010 [141]	To determine if the IDEATel project would help lower LDL-C cholesterol for older, ethnically diverse older adults.	The IDEATel project increased statin use and lowered LDL-C levels.
Glycemic control and health disparities in older ethnically diverse underserved adults with diabetes: five-year results from the Informatics for Diabetes Education and Telemedicine (IDEATel) study.	Weinstock et al., 2011 [142]	To discuss the possible benefits of telemedicine intervention	Telemedicine intervention can reduce racial and ethnic disparities in glycemic control for older and underserved adults; Hispanics had the greatest improvement
Goal setting using telemedicine in rural underserved older adults with diabetes: experiences from the informatics for diabetes education and telemedicine project.	West et al., 2010 [143]	To describe using IDEATel to set goals for behavioral change.	Out of all the rural patients, the elderly participants had the most difficulty using the computer. Overall, televisits were found to be a successful way to help improve diabetes self management
Does entertainment-education work with Latinos in the United States? Identification and the effects of a	Wilkin et al., 2007 [144]	Examines the possible benefits of providing health information on a Telenova	Education through use of a telenovela works

telenovela breast cancer storyline.			
The effect of an educational intervention on promoting breast self-examination in older African American and Caucasian women.	Wood et al., 2002 [145]	To determine the effectiveness of an age and race sensitive, self-monitored, video breast health kit for increasing knowledge about breast cancer.	The age and race sensitive video intervention was successful at increasing knowledge about both breast cancer risk and breast cancer screening.
Tribal connections health information outreach: results, evaluation, and challenges.	Wood et al., 2003 [146]	To discuss the implementation of the Pacific Northwest Regional Medical Library (PNRLM) which was created to improve tribal access to health information on the internet.	The project showed how important the involvement and empowerment of the community is for a successful outreach.
The health impact of resolving racial disparities: An analysis of US mortality data	Woolf et al., 2004 [147]	To determine if the focus on improving and including technology of care (aka drugs and devices) is hindering the process of reducing mortality disparities.	Achieving equity between African Americans and Whites would reduce mortality more than technological advances
Text messaging for HIV prevention with young Black men: formative research and campaign development	Wright et al., 2011 [148]	To test the feasibility of using text messages as an HIV prevention method for young African-American Men	The focus groups found that the men were open to text messages and suggested that humor be used and that the messages not be directed only at African-Americans as they felt like the senders were engaging in stereotypes
Bilingual health literacy assessment using the Talking Touchscreen/la Pantalla Parlanchina: Development and pilot testing.	Yost et al., 2009 [149]	To determine the effectiveness of commercials about fruits and vegetables can have an impact on children's preferences for certain fruits and vegetables	The talking touchscreen is expected to help improve health literacy and will be evaluated after it is tested on 1200 primary care patients
